# Genome-Wide Identification and Functional Analysis of *CLAVATA3/EMBRYO SURROUNDING REGION-RELATED* (*CLE*) in Three *Populus* Species

**DOI:** 10.3390/ijms26051944

**Published:** 2025-02-24

**Authors:** Zheng Li, Feng-Xin Chen, Ming-Ming Li, Xian-Li Tang, Yu-Qi Liu, Meng-Bo Huang, Hao-Qiang Niu, Chao Liu, Hou-Ling Wang, Xin-Li Xia, Wei-Lun Yin

**Affiliations:** State Key Laboratory of Tree Genetics and Breeding, National Engineering Research Center of Tree Breeding and Ecological Restoration, College of Biological Sciences and Technology, Beijing Forestry University, Beijing 100083, China; zhengli@bjfu.edu.cn (Z.L.); chenfx@bjfu.edu.cn (F.-X.C.); limingming@bjfu.edu.cn (M.-M.L.); tangxl@bjfu.edu.cn (X.-L.T.); liuyuqi0401@bjfu.edu.cn (Y.-Q.L.); hmb425069@bjfu.edu.cn (M.-B.H.); niuhaoqiang@bjfu.edu.cn (H.-Q.N.); liuchao1306@bjfu.edu.cn (C.L.); whling@bjfu.edu.cn (H.-L.W.)

**Keywords:** plant peptides, bioinformatic analysis, drought resistance, *Populus trichocarpa*, *Populus tomentosa*, *Populus alba* × *Populus glandulosa*

## Abstract

Intercellular communication mediated by CLAVATA3/EMBRYO SURROUNDING REGION-RELATED (CLE) peptides and their receptors is crucial for plant development and environmental adaptation. In this study, 45 and 89 *CLEs* were identified in *Populus tomentosa* and *Populus alba* × *Populus glandulosa*, respectively, and, together with the 52 *CLEs* in *Populus trichocarpa*, the chromosome localization, gene and protein characteristics, collinearity and gene duplication events, cis-acting regulatory elements in promoters and evolutionary relationships of *CLEs* in these three poplar species were analyzed. The *CLEs* of three poplar species were divided into four subfamilies. Among them, the *CLEs* in subfamilies I, II and IV were A-type *CLEs*, while those in subfamily III were B-type *CLEs*. During the evolutionary process of poplar, the selection pressure faced by whole-genome duplication or segmental duplication was purifying selection, and the duplication events led to the expansion of the *CLE* family in poplar. The exogenous addition of a certain concentration of poplar CLE13 peptides inhibits the root growth of *Arabidopsis thaliana* and poplar and simultaneously reduces the expression levels of *ARFs* and *LBDs* in the roots. In addition, drought stress induces the expression of *PtrCLE13A*. The overexpression of *preCLE13A* significantly enhances the osmotic and drought tolerance in *Populus tomentosa*. These results have provided valuable information for further research on the molecular mechanisms of CLE peptide signaling pathways in the woody model plant poplar regarding plant growth and stress resistance.

## 1. Introduction

Intercellular communication is essential for the development and environmental adaptation of plants. Besides systemic hormones, small RNAs and gases such as ethylene and nitric oxide, certain plant peptides can also serve as signaling molecules that bind to specific receptors to complete the intercellular signaling in plants.

The majority of the characterized plant peptides reported are derived from nonfunctional precursors or functional precursors; are not derived from a precursor protein such as the plant peptides synthesized directly from small open reading frames (sORFs); or are encoded by primary products of microRNAs [1,2]. A typical class of post-translationally modified peptides from nonfunctional precursors in plants is encoded by the *CLAVATA3/EMBRYO SURROUNDING REGION-RELATED* (*CLE*) gene family. CLAVATA 3 (CLV3) was the first CLE peptide to be identified, and loss of function of CLV3 resulted in enlargement of shoot apical meristem (SAM) [3]. According to the different effects on plant growth and development, *CLEs* are mainly divided into two types, A and B, in *Arabidopsis thaliana* [4]. A-type *CLEs* affect the activity of stem cells in plant roots or shoots, and B-type *CLEs* promote the proliferation of procambial cells and inhibit their differentiation into vessels [5]. Most *CLE* genes encode a propeptide composed of an N-terminal signal peptide, a central variable region and a C-terminal CLE conserved domain, then signal peptides of the propeptide are cut during transport to the apoplast via secretory pathways, and variable regions are excised by serine peptidase and carboxypeptidase. After that, a peptide consisting of a CLE conserved domain containing 12–13 amino acids is formed [6]. Finally, the fourth and seventh proline residues of the peptide are usually hydroxylated, and the seventh Pro^Hyp^ residue is modified with three residues of L-arabinose [7,8]. The post-translationally modification can enhance the binding ability between CLE peptide and its receptor [9].

TRACHEARY ELEMENT DIFFERENTIATION INHIBITORY FACTOR (TDIF), a dodecapeptide encoded by B-type *CLEs*, binds to the TDIF RECEPTOR/PHLOEM INTERCALATED WITH XYLEM (TDR/PXY) receptor to perform its function in promoting (pro) cambium cell proliferation, inhibiting xylem cell differentiation and increasing the number of lateral roots. On the one hand, TDIF peptides from phloem bind to the (pro) cambium receptor TDR/PXY, promoting (pro) cambium cell proliferation by upregulating the expression of *WUSCHEL-RELATED HOMEOBOX 4* (*WOX4)* and *WOX14* genes, which are related to (pro) cambium cell division [10,11]. On the other hand, the TDIF-TDR/PXY signaling pathway activates GSK3s from the SKⅠ and SKⅡ subfamilies of the Glycogen Synthase Kinase 3 (GSK3) family, thereby inhibiting the BRⅠ1-EMS-SUPPRESSOR 1 (BES1) transcription factor that actively regulates xylem cell differentiation and thus inhibiting xylem cell differentiation [12,13]. In addition, BRASSINOSTEROID INSENSITIVE 2 (BIN2), a member of the SKII subfamily of the GSK3 family, is phosphorylated directly via the TDIF-TDR/PXY signaling module, then BIN2-mediated phosphorylation of AUXIN RESPONSE FACTOR 7 (ARF7) and ARF19 inhibited their interaction with AUX/IAAs and subsequently enhanced transcriptional activity to their target genes *LATERAL ORGAN BOUNDARIES-DOMAIN 16* (*LBD16*) and *LBD29* [14]. Therefore, the TDIF-TDR-BIN2 signaling cascade in the xylem-pole pericycle increases the number of lateral roots.

Poplar TDIF signaling pathway affects cambium cell division and vascular development. PtrTDIF peptides (HEVP^Hyp^SGP^Hyp^NPISN) and PtrTDIF-like peptides (HEVP^Hyp^SGP^Hyp^NPESN) are encoded by B-type *CLEs* in *Populus trichocarpa*. Although PtrTDIF and PtrTDIF-like peptides differ only in the 10th amino acid, they both affect plant vascular development [15]. Moreover, overexpression of *PttCLE41B* and its receptor gene *PttPXY* in hybrid aspen, either separately or at the same time, resulted in abnormal vascular tissue development and plant dwarfing. When the two genes were correctly overexpressed in the poplar cambium region, the wood formation rate of transgenic plants was increased by two times compared with the wild type [16]. Overexpression of *PttCLE41b*, *PttCLE41c* and *PttCLE41d* in hybrid aspen can promote the expression of *PttWOX4*, which is related to (pro) cambium cell division, and then actively regulate cambium cell division to promote secondary growth [17].

The poplar TDIF signaling pathway can also interact with the auxin signaling pathway to influence the development of poplar roots. Both the application of exogenous TDIF/TDIFL peptides and the overexpression of TDIF-related genes can positively regulate the initiation and elongation of lateral roots (LRs) in *P. tremula* × *P. alba*. The reasons are as follows: On the one hand, the overexpression of the *PtTDIF2*/*PtTDIFL2* genes can enhance the synthetic ability of indole-3-acetic acid (IAA) in the shoot tips of poplar, and the auxin flux moves from the stems to the tip of the primary root through the vascular system, resulting in an increase in the auxin content in the LRs of poplar. On the other hand, the overexpression of the *PtTDIF2*/*PtTDIFL2* genes can upregulate the expression levels of auxin-related LR initiation marker genes such as *GATA transcription factor 23* (*GATA23*), *LBD16* and *LBD29* in the roots of poplar to promote the development of LRs [18].

The poplar TDIF signaling pathway retards internodal elongation and enhances leaf venation through interacting with auxin and gibberellins (GAs). In one respect, overexpression of *PtTDIF2*/*PtTDIFL2* genes in hybrid poplar inhibits internodal elongation by upregulating the expression of *gibberellin 2-oxidase* (*GA2ox*) and *gibberellin 20-oxidase* (*GA20ox*) genes, thus reducing the levels of endogenous GAs. In another respect, overexpression of *PtTDIF2*/*PtTDIFL2* induced a more complex vein pattern in poplar leaves, which might be caused by the induced expression of *WOX4*, *WOX13* and multiple *PIN* genes [19].

The poplar A-type CLE signaling pathway plays a role in secondary growth. The signaling mediated by the CLE20 peptide located in the vascular cambium of *P. trichocarpa* and its receptor CLV2 inhibits the activity of the vascular cambium and secondary development of poplar by downregulating the expression levels of genes related to cell division such as *RESPONSE REGULATOR 5* (*RR5*) and *Cyclin A1* (*CycA1*), genes related to secondary cell development in poplar such as *WOX4*, *class III HD-ZIP 7* (*HB7*), *LBD1* and *WOOD-ASSOCIATED NAC TRANSCRIPTION FACTOR 1B* (*WND1B*) which are related to secondary cell development in poplar [20]. In addition, the RNAi poplar plants of the *PttCLE47* gene exhibited traits such as a reduction in the number of cell layers in the cambium region, a narrow secondary xylem region, slow apical growth and a decrease in leaf area by suppressing the expression of genes including *HB4*, *HB7*, *WOX4* and *PXY* [21].

Poplar is one of the forest trees with high ecological and economic values. There is little research on the identification and functional studies of *CLE* genes in poplar. This study conducts a whole-genome identification of *CLE* genes in *P. tomentosa* and *P. alba* × *P. glandulosa* ‘84K’. Meanwhile, the chromosome location, gene structure, protein characteristics, cis-acting elements of promoters and intragenomic and intergenomic duplications of the *CLE* genes of the three poplar species were studied. In addition, the effects of the *PtrCLE13A* gene on plant development and drought resistance were revealed through in vitro peptide treatment and transgenic methods. These research results not only provide necessary resources for further exploring the molecular mechanisms of intercellular communication mediated by CLE peptides and their receptors in poplar but also help create poplar germplasm with good drought resistance through genetic engineering.

## 2. Results

### 2.1. Identification and Chromosomal Distribution of CLE Genes in Three Poplar Species

To name each member of the *CLE* gene family identified from the genomes of *P. tomentosa* and *P. alba* × *P. glandulosa*, we constructed phylogenetic trees based on the CLE motifs, separately for comparisons between *P. trichocarpa* and *P. tomentosa* as well as between *P. trichocarpa* and *P. alba* × *P. glandulosa* (Supplementary Figure S1A,C).

Finally, in total, 45 and 89 *CLE* genes were identified in *P. tomentosa* and *P. alba* × *P. glandulosa* genomes, respectively, and the genome of *P. trichocarpa* contains 52 *CLE* genes [20]. The *CLE* genes of *P. trichocarpa*, *P. tomentosa* and *P. alba* × *P. glandulosa* encode 39, 23 and 40 mature CLE peptides, respectively. The distribution of *CLE* genes of three poplar species on chromosomes is shown in Supplementary Figure S2. The *CLEs* of *P. trichocarpa* are unevenly distributed on chromosomes except Chr06, Chr07 and Chr18, and there are relatively larger numbers of *CLEs* on Chr01, Chr10 and Chr08, with 9, 7 and 6, respectively, while the number of *CLEs* on other chromosomes ranges from 1 to 4 (Supplementary Figure S2A). There is no distribution of *CLEs* on the chromosomes A02, A05, A06, A07, A14, A16, A18, D05, D06, D07, D14, D16 and D18 of *P. tomentosa*, and the number of *CLEs* distributed on each of the other chromosomes ranges from 1 to 4 (Supplementary Figure S2B). There are no *CLEs* on chromosomes A07, A18, G07 and G18 of *P. alba* × *P. glandulosa*, and on the other chromosomes, the number of *CLEs* on chromosomes A01, A08, A10, A11, G01 and G08 ranges from 5 to 7, and there are 1 to 4 *CLEs* on the remaining chromosomes (Supplementary Figure S2C). There are commonalities in the chromosomal distribution of *CLEs* among the three poplar species: (i) *CLEs* are absent on chromosomes 7 and 18 in all three poplar species; (ii) the number of *CLEs* on at least 80% of the chromosomes ranges from 1 to 4 in each type of poplar tree; and (iii) the number of *CLEs* on chromosome 1, which is the longest chromosome in all three poplar species, is also the largest. The number of *CLEs* on each chromosome is not related to the length of the chromosome, because in all three poplar species, there are situations where the number of *CLEs* on shorter chromosomes is greater than that on longer chromosomes.

### 2.2. Characteristics of CLE Gene Structures and Precursor Proteins in Three Poplar Species

There are both similarities and differences in the structures of *CLE* genes and CLE precursor protein domains among the three poplar species. Most *CLE* genes in *P. trichocarpa* and *P. alba* × *P. glandulosa* have no introns (71.15% in *P. trichocarpa* and 75.28% in *P. alba* × *P. glandulosa*), while most (84.44%) *CLE* genes in *P. tomentosa* have introns, and 80.77%, 40.45% and 24.44% of the gene members have untranslated regions in the *CLE* gene families of *P. trichocarpa*, *P. alba* × *P. glandulosa* and *P. tomentosa*, respectively. The CLE precursor proteins of each kind of poplar have a total of five motifs. Moreover, all the CLE precursor proteins of the three kinds of poplars possess motif 1, while the distribution of other types of motifs is uneven. Specifically, in *P. trichocarpa*, 92.31% of the CLE precursor proteins contain motif 2, while only PtrCLE41B, PtrCLE44A and PtrCLE44B have motif 3, only PtrCLE45A and PtrCLE45B contain motif 4 and only PtrCLE41C and PtrCLE41D have motif 5. In addition, eight PtoCLEs have motif 2, six PtoCLEs have motif 3, four PtoCLEs have motif 4, and seven PtoCLEs have motif 5. Also, 55.06% of the CLE precursor proteins in *P. alba* × *P. glandulosa* have motif 5, while only eight PagCLEs have motif 2, thirteen PagCLEs have motif 3, and four PagCLEs have motif 4. See the Supplementary Figure S3 for details.

Each *CLE* gene encodes a pre-propeptide consisting of a signal sequence that directs the protein through the secretory pathway, a highly variable region, and a conserved motif near the C-terminus called the CLE motif (Supplementary Figure S4). These *CLEs* encoded a wide range of amino acids, from 53 (PagCLE13.2) to 226 (PtoCLE45.3), and the average is 101. The molecular weights (Mw) of CLE pre-propeptides in the three poplar species varied between 6.099 kDa and 26.222 kDa, and the average is 11.305 kDa. Ten genes encoding CLE pre-propeptides with a molecular weight > 15 kDa and five genes encoding PtoCLEs with a molecular weight > 20 kDa were identified. The isoelectric points (pI) of all CLE pre-propeptides range from 5.37 to 12.01, with a mean value of 9.55. There were 39% CLE pre-propeptides that do not have a signal peptide cleavage site in three poplar species, indicating that these may affect the formation of mature peptide (Supplementary Tables S1–S3).

### 2.3. Evolutionary Relationships of CLE Genes and Characteristics of CLE Motifs in Three Poplar Species

**Figure 1:**
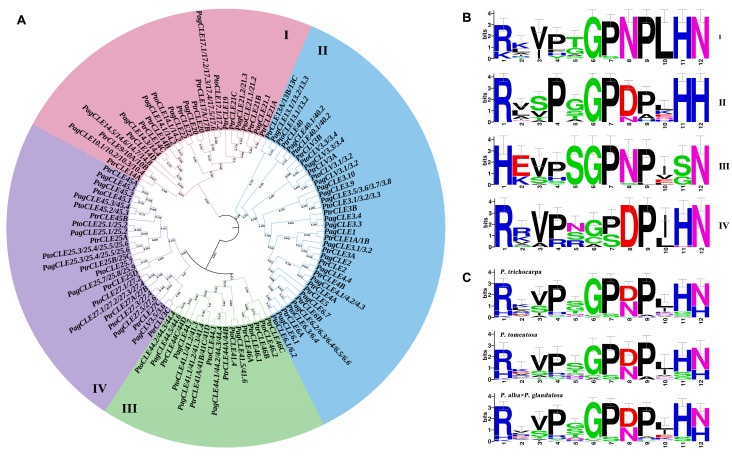
The phylogenetic tree was constructed using MEGA7 with the neighbor-joining method. (**A**) The phylogenetic tree of *CLEs* among *P. trichocarpa*, *P. tomentosa* and *P. alba* × *P. glandulosa*. (**B**) The weblogo represents CLE motifs (12 conserved amino acids) of four groups. (**C**) The weblogo represents CLE motifs (12 conserved amino acids) of *P. trichocarpa*, *P. tomentosa* and *P. alba* × *P. glandulosa*. The website of weblogo is https://weblogo.berkeley.edu/logo.cgi (accessed on 4 March 2024).

According to CLE motifs, all the *CLE* genes in *P. trichocarpa*, *P. tomentosa* and *P. alba* × *P. glandulosa* genomes were also classified into subgroups Ⅰ to Ⅳ (Figure 1A). Subgroups Ⅰ to Ⅳ contained 44 (6 *PtoCLEs*, 15 *PtrCLEs* and 23 *PagCLEs*), 63 (15 *PtoCLEs*, 16 *PtrCLEs* and 32 *PagCLEs*), 34 (10 *PtoCLEs*, 11 *PtrCLEs* and 13 *PagCLEs*) and 45 (10 *PtrCLEs*, 14 *PtoCLEs* and 21 *PagCLEs*) *CLEs*, respectively. The CLE motif weblogos were drawn for each group to display the conserved sequences. In any phylogenetic tree, the following characteristics of CLE motifs can be identified in different subgroups: (i) the first residue of the CLE motif of subgroups Ⅰ, Ⅱ and Ⅳ mainly appeared as R (Arg), while the subgroup Ⅲ was presented as H (His); (ii) the CLE motif of subgroups Ⅰ, Ⅲ and Ⅳ almost terminated with the residue N (Asn), whereas subgroup Ⅱ typically concluded with H (His); and (iii) the residues at the sixth, seventh, eighth, and ninth bits of the CLE motif in subgroups Ⅰ and Ⅲ are more frequently represented as G (Gly) P (Pro) N (Asn) P (Pro), while subgroups Ⅱ and Ⅳ exhibited a higher frequency of the G (Gly) P (Pro) D (Asp) P (Pro) form (Figure 1B and Supplementary Figure S1B,D). From the evolutionary relationship and the characteristics of CLE conserved domain, it can be concluded that the genes in the Ⅲ subgroup are B-type *CLEs*, while the genes in the other three subgroups are A-type *CLEs*. According to the weblogos of the CLE conserved domains of three poplar species, it can be concluded that the CLE motif of these three poplar species is R(H)XVPXGPD(N)PLHN(H) (Figure 1C).

### 2.4. Analysis of Collinearity, Duplication and Ka/Ks Values of the CLE Gene Families

**Figure 2:**
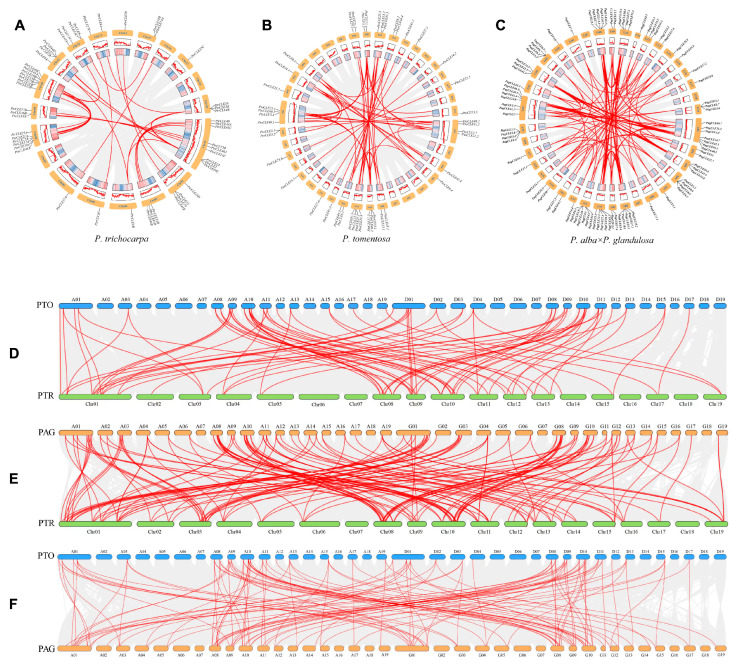
The collinear relationships of *CLE* genes within and between species of *P. trichocarpa*, *P. tomentosa* and *P. alba* × *P. glandulosa*. (**A**–**C**) respectively represent the distribution of the intra-genomic collinear pairs of *CLE* genes within *P. trichocarpa*, *P. tomentosa* and *P. alba* × *P. glandulosa* on the chromosomes, and the orange boxes represent chromosomes. (**D**) *P. tomentosa* and *P. trichocarpa*; (**E**) *P. alba* × *P. glandulosa* and *P. trichocarpa*; (**F**) *P. tomentosa* and *P. alba* × *P. glandulosa*. The gray lines: the collinearity of the whole genome among poplar. The red lines: the collinearity of *CLE* gene pairs. The collinearity analysis was conducted using TB tools, and the website is https://github.com/CJ-Chen/TBtools (accessed on 16 March 2024).

In order to conduct an in-depth study on the evolutionary relationships of *CLEs* in *P. trichocarpa*, *P. tomentosa* and *P. alba* × *P. glandulosa*, the analysis was carried out on the intragenomic and intergenomic gene duplication events among all the *CLEs* of these three poplar species. In *P. trichocarpa*, *P. tomentosa* and *P. alba* × *P. glandulosa*, 24, 33 and 92 pairs of intragenomic homologous gene pairs of *CLEs* were identified, respectively, and the number of homologous gene pairs in the genome of *P. alba* × *P. glandulosa* is 3.83 times and 2.79 times that of *P. trichocarpa* and *P. tomentosa*, respectively (Figure 2A–C and Supplementary Table S4). Moreover, the homologous gene pairs of *P. trichocarpa* are mainly concentrated on chromosomes Chr01, Chr08 and Chr10; the homologous gene pairs of *P. tomentosa* are mainly concentrated on chromosomes A01, A03, A04, D01, D03, D04, A08-A11 and D08-D11; and the homologous genes of *P. alba* × *P. glandulosa* are mainly concentrated on chromosomes A01, A03, G01, G03, A08-A10 and G08-G10 (Figure 2A–C). There are 70, 137 and 122 pairs of collinear pairings between *CLEs* and homologous genes among *P. trichocarpa* and *P. tomentosa*, *P. trichocarpa* and *P. alba* × *P. glandulosa* and *P. tomentosa* and *P. alba* × *P. glandulosa*, respectively (Figure 2D–F and Supplementary Table S4). The collinearity analysis shows that all the duplicated *CLE* gene pairs are the result of whole-genome duplication or segmental duplication (Supplementary Table S4).

The Ka/Ks ratio represents the selective pressure faced by gene duplication. Generally speaking, a Ka/Ks < 1 indicates purifying selection, a Ka/Ks = 1 indicates neutral evolution and a Ka/Ks ratio > 1 indicates directional selection (37). The Ka/Ks ratios of most *CLE* homologous gene pairs in the genomes of the three poplar species are <1. In particular, in *P. trichocarpa*, there is no Ka/Ks value ≥ 1, and only six gene pairs have Ka/Ks values > 1 (Supplementary Table S5). Similarly, the Ka/Ks ratios of most *CLE* gene pairs among the genomes of the three poplar species are also <1. However, there are more gene pairs with Ka/Ks values > 1 among the genomes than within the genomes, and there are 27 pairs in total (Supplementary Table S6). In conclusion, the *CLEs* of the three poplar species mainly underwent purifying selection during the evolutionary process, and there were also a few results of directional selection, such as the 33 gene pairs with Ka/Ks > 1 mentioned above.

### 2.5. Analysis of Cis-Acting Regulatory Elements in Promoters of CLE Genes in Three Poplar Species

**Figure 3:**
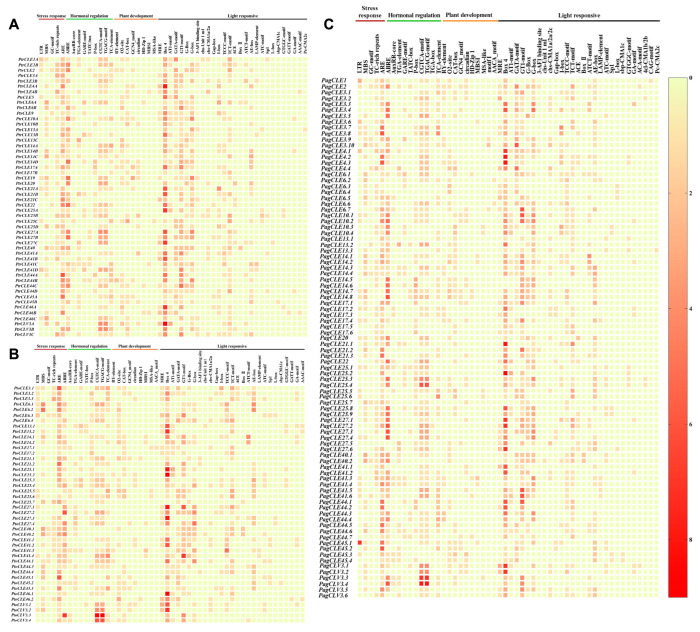
Analysis of cis-acting regulatory element numbers of *CLE* genes of *P. trichocarpa* (**A**), *P. tomentosa* (**B**) and *P. alba* × *P. glandulosa* (**C**). The left-most column of each heatmap shows the gene names. Above the heatmap represent different types of CAREs (the CAREs corresponding to the area below the brown horizontal line are related to stress response; the CAREs corresponding to the area below the green horizontal line are related to hormone regulation; the CAREs corresponding to the area below the orange horizontal line are related to plant development; and the CAREs corresponding to the area below the black horizontal line are related to light response). In the heatmap, the color intensity of the color blocks indicates the number of CAREs (as shown by the color strip on the far right of the entire figure, the number increases from light yellow to dark red). All the heatmaps were generated by GraphPad Prism 8.

In total, 50, 50 and 54 types of cis-acting regulatory elements (CAREs) were observed in the promoters of *CLE* genes in *P. trichocarpa*, *P. tomentosa* and *P. alba* × *P. glandulosa*, respectively. These were identified as four functional types: stress response, hormonal regulation, plant development and light responsive (Figure 3). The number of each CARE in each poplar species and the total amount of each type of element in each poplar species was counted, and the corresponding percentage was calculated (Supplementary Figures S5 and S6). Among them, CAREs involved in light responsiveness accounted for 50.43–52.67% of the total elements, followed by CAREs related to hormonal regulation (25.06–27.73%), stress-related CAREs (14.63–15.51%) and those related to plant development (7.07–7.34%) (Supplementary Figure S6(A1,B1,C1)). Among the three poplar species, Box 4, which is part of a conserved DNA module associated with light response, exhibited the highest proportion (24.56–27.15%) of CAREs related to light response (Supplementary Figure S6(A5,B5,C5)). Among the CAREs associated with light response in *CLE* genes of three poplar species, chs-CMA2c, ACA-motif, 4cl-CMA1b/2b and CAG-motif elements are specific to *P. alba* × *P. glandulosa*, and GATT-motif is exclusively found in *P. trichocarpa* and *P. tomentosa*. Additionally, *P. tomentosa* does not contain the Pc-CMA2c element (Figure 3 and Supplementary Tables S7–S9). Among the CAREs involved in hormonal regulation, the higher proportions were found for the ABRE, CGTCA and TGACG elements (Supplementary Figure S6(A3,B3,C3)). The CAREs related to stress response showed higher proportions in both ARE and MBS elements (Supplementary Figure S6(A2,B2,C2)). The O2-site and CAT-box elements accounted for a relatively high proportion of CAREs related to plant development (Supplementary Figure S6(A4,B4,C4)). Among the CAREs linked to plant development in *CLE* genes of three poplar species, the motif I element is specific to *P. alba* × *P. glandulosa*, and only *P. trichocarpa* lacks the AACA_motif element (Figure 3 and Supplementary Tables S7–S9). The annotations of CAREs can be found in Supplementary Table S10.

Additionally, we observed distinct types of CAREs in the promoter region of each *CLE* gene across the three poplar species, with multiple instances of the same type of CAREs in the promoter region of most *CLE* genes. Among the *CLE* genes of the three poplar species that contain stress response-related CAREs in their promoters, no *CLE* gene simultaneously contains all the CAREs associated with stress response, but *PtoCLE27.2* and *PagCLE27.5* do not contain them. Meanwhile, a total of 37, 30 and 70 *CLE* genes containing two to four stress response-related CAREs in the promoter were found in *P. trichocarpa*, *P. tomentosa* and *P. alba* × *P. glandulosa*, respectively. In addition, fourteen *PtrCLEs*, eleven *PtoCLEs* and thirteen *PagCLEs* only contain ARE element; *PtrCLE14D* and three *PagCLEs* only contain TC-rich repeats element; *PtoCLE25.5* and *PtoCLE27.1* only contain LTR element; and *PagCLE3.5* only contains MBS element (Supplementary Figure S7A,C,E). Among the *CLE* genes of the three poplar species that contain hormonal regulation-related CAREs in their promoters, a total of two, four *PtoCLEs* and four *PagCLEs* containing CAREs involved in all five types of hormonal regulation (Auxin, SA, ABA, MeJA and GA) in the promoter were found in *P. trichocarpa*, *P. tomentosa* and *P. alba* × *P. glandulosa*, respectively, but *PtrCLE46B*, *PtoCLE44.4*, *PtoCLE46.2* and *PagCLE6.4* do not have any type of hormonal regulation-related CAREs. *PtrCLE10B*, two *PtoCLEs* and three *PagCLEs* only have elements that respond to GA; three *PtrCLEs* only have elements that respond to ABA; *PtoCLV3.2*, two *PtrCLEs* and two *PagCLEs* only have elements that respond to MeJA; and two *PagCLEs* only have elements that respond to SA (Supplementary Figure S7B,D,F).

### 2.6. preCLE13 Expression Pattern Analysis in Specific Tissues and Dehydration Stress

**Figure 4:**
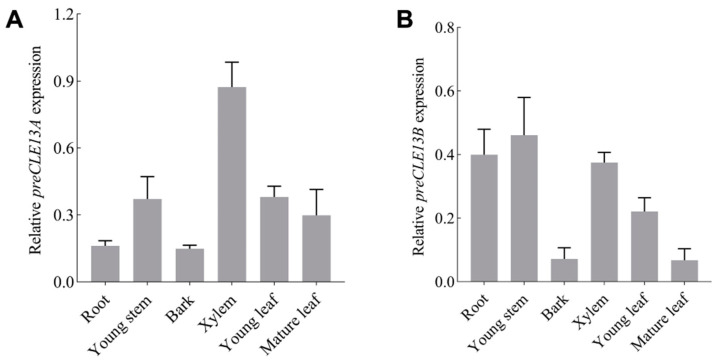

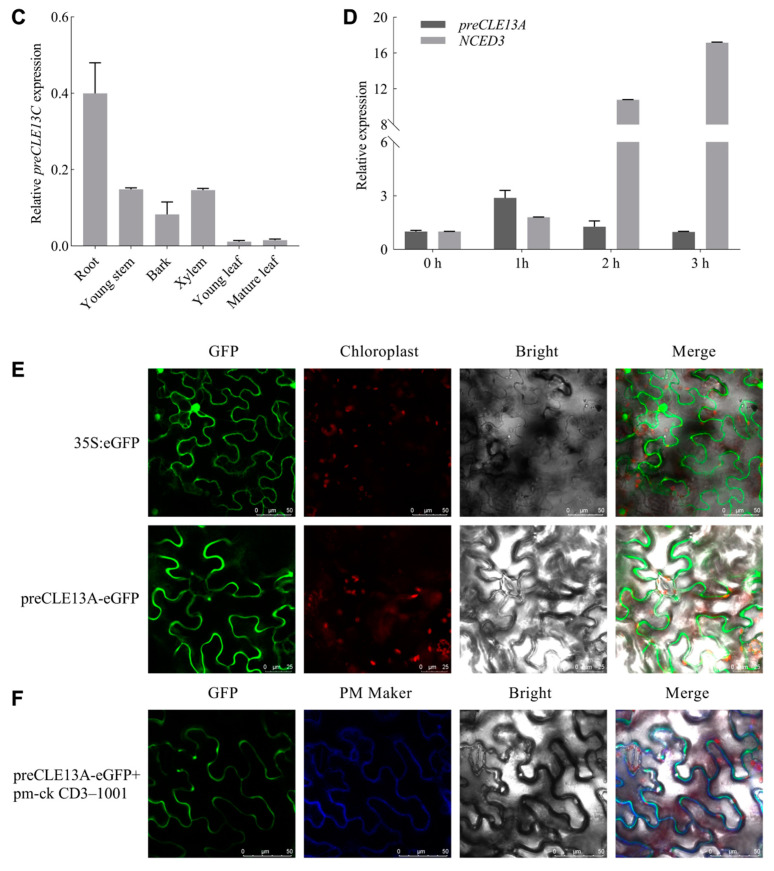
The expression patterns of *preCLE13* and subcellular localization of pre-propeptide of PtrCLE13A. (**A**–**C**) Expression patterns of *preCLE13A*, *preCLE13B* and *preCLE13C* in different tissues of *P. trichocarpa*. (**D**) Expression of *preCLE13A* and *NCED3* in response to dehydration treatment. Three-month-old *P. trichocarpa* with good growth was used as the material for RT-qPCR analysis, and each experiment was based on three biological replicates of each sample and three technical replicates of each biological replicate. (**E**) The 35Spro:eGFP and 35Spro:preCLE13A-eGFP were transiently infiltrated in *N. benthamiana* leaves. (**F**) The 35Spro:preCLE13A-eGFP construct was transiently infiltrated in *N. benthamiana* leaves with pm-ck CD3-1001 construct as PM (plasma membrane) marker. Microscopic images contain green fluorescence field, chloroplast field, bright field and merged microscope images. Bars = 50 µm. Data are means ± SE.

RT-qPCR was applied to determine the relative transcription levels of *preCLE13* (*PtrCLE13*) in various tissues in *P. trichocarpa*. The results revealed that *preCLE13A* (*PtrCLE13A*) was preferentially expressed in xylem, young stems, young leaves and mature leaves in *P. trichocarpa* (Figure 4A). The *preCLE13B* (*PtrCLE13B*) had high expression levels in young stems, roots, xylem and young leaves of *P. trichocarpa* (Figure 4B). The expression of *preCLE13C* (*PtrCLE13C*) in *P. trichocarpa* roots, young stems and xylem was higher than that in young leaves and mature leaves (Figure 4C).

The predictive analysis through PlantCARE showed that there were MBS and ABRE elements in the promoter of *PtrCLE13A* (Figure 3A). In addition, the gene expression patterns of *preCLE13* from the website (https://plantgenie.org/ (accessed on 20 March 2024) indicated that only *preCLE13A* was induced to express in *P. trichocarpa* leaves under drought (Supplementary Figure S8A). As determined by RT-qPCR analysis of *P. trichocarpa* leaves treated with dehydration for the indicated times, we validated the transcriptional induction of the *preCLE13A* gene by dehydration stress, and the expression level of *NCED3*, as a marker gene in dehydration stress, was increased obviously with the prolongation of dehydration time (Figure 4D).

### 2.7. Subcellular Localization of Pre-Propeptide of PtrCLE13A

To determine the subcellular localization of pre-propeptide of PtrCLE13A (preCLE13A), we transiently expressed the preCLE13A-GFP (green fluorescent protein, GFP) construct in *Nicotiana benthamiana* leaves. The fluorescence signals were observed in the epidermal cells. The results showed that 35S:eGFP was visualized in the whole cells without specific localization; however, the preCLE13A-eGFP fusion protein was observed specifically around the cytomembrane (Figure 4E). Moreover, we transiently co-infiltrated preCLE13A-GFP and pm-ck CD3-1001 (cyan fluorescent protein, CFP) constructs into *N. benthamiana* leaves. The results showed that preCLE13A-eGFP and pm-ck CD3-1001 were distinctly detected on the membrane (Figure 4F).

### 2.8. Phenotypic Analysis of A. thaliana and P. tomentosa Treated with PtrCLE13 Peptide

**Figure 5:**
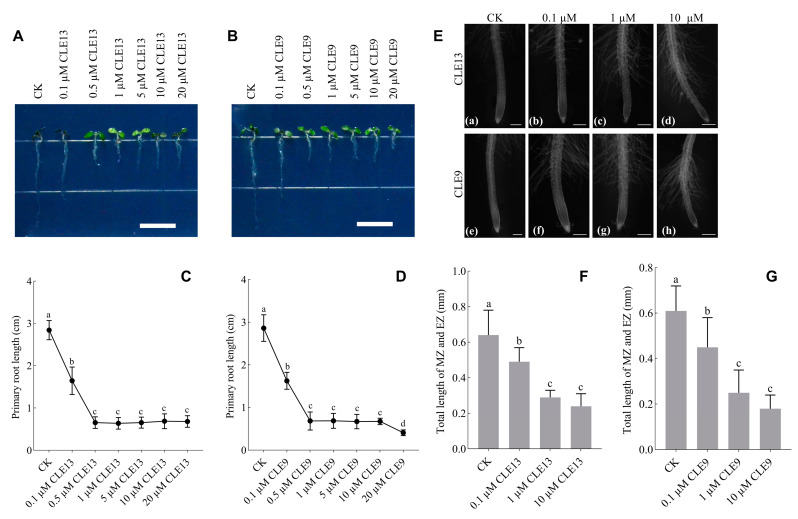
PtrCLE13 and CLE9 peptides inhibit root meristem activity in *A. thaliana*. (**A**–**D**) Inhibitory effects on root growth of *Arabidopsis* seedlings after 10-day treatment with different concentrations of PtrCLE13 and CLE9 peptides. Bar = 1 cm. (**E**–**G**) Length of meristematic zone (MZ) and elongation zone (EZ) of *Arabidopsis* root tips after 10-day treatment with 0 [(**a**,**e**)], 0.1 µM [(**b**,**f**)], 1 µM [(**c**,**g**)] and 10 µM [(**d**,**h**)] PtrCLE13 and CLE9 peptide concentrations, respectively. Bar = 0.125 mm. Data are presented as means ± SD (*n* = 30 for (**C**,**D**,**F**,**G**)). Different lowercase letters indicate a significant difference at *p* < 0.05 based on ANOVA.

The results of the multi-sequence alignment revealed that the mature peptides containing the CLE domain generated from preCLE13A, preCLE13B and preCLE13C were identical, and all of the sequences were RLVPTGPNPLHH (Supplementary Figure S3 and Table S1). We treated *A. thaliana* and *Populus* micro-propagated seedlings with synthetic PtrCLE13 peptide. Meanwhile, synthetic CLE9 peptide reported to inhibit plant root growth was used as a positive control [22]. The primary root length of *Arabidopsis* seedlings exhibited an initial decrease followed by stabilization as the concentration of PtrCLE13 peptide increased, mirroring the trend observed in CLE9 peptide treatment (Figure 5A–D). To examine how PtrCLE13 peptide affects root growth, we observed *Arabidopsis* root tips untreated and treated with PtrCLE13 peptide. The results showed that the lengths of the meristematic zone (MZ) and elongation zone (EZ) in the root tips was shorter. In detail, the application of 0.1 μM, 1 μM and 10 μM of PtrCLE13 peptide reduced the total length of the MZ and EZ at the root tips of *A*. *thaliana* by 23.44%, 54.69% and 62.50%, respectively, compared with the control group. Moreover, the application of 0.1 μM, 1 μM and 10 μM of CLE9 peptide decreased the total length of the MZ and EZ at the root tips of *A*. *thaliana* by 26.23%, 59.02% and 70.49%, respectively, relative to the control group. The above results indicate that RAM activity was inhibited (Figure 5E–G).

**Figure 6:**
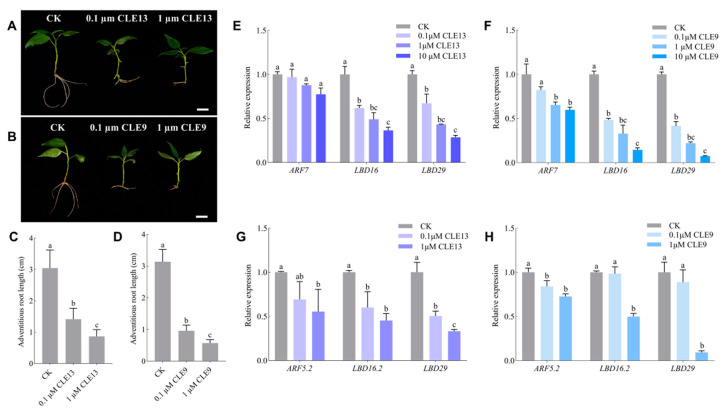
PtrCLE13 and CLE9 peptides exert inhibitory effects on root growth in *P. tomentosa* and *A. thaliana* by downregulating the expression of *ARF* and *LBD* genes. (**A**,**B**) Root morphology of *P. tomentosa* after two-week treatment with 0 (left), 0.1 (middle) and 1 µM (right) PtrCLE13 and CLE9 peptides concentrations. Bar = 1 cm. (**C**,**D**) The root length of *P. tomentosa* was measured after three-week treatment with different concentrations of PtrCLE13 and CLE9 peptides. (**E**,**F**) Expression levels of *AtARF7*, *AtLBD16* and *AtLBD29* in *Arabidopsis* roots after 12-day treatment with different concentrations of PtrCLE13 and CLE9 peptides, respectively. (**G**,**H**) Expression levels of *PtoARF5.2*, *PtoLBD16.2* and *PtoLBD29* in *P. tomentosa* roots after three-week treatment with different concentrations of PtrCLE13 and CLE9 peptides, respectively. Data are presented as means ± SD (*n* = 20 for (**C**,**D**) or *n* = 3 for (**E**–**H**). Different lowercase letters indicate a significant difference at *p* < 0.05 based on ANOVA.

**Figure 7:**
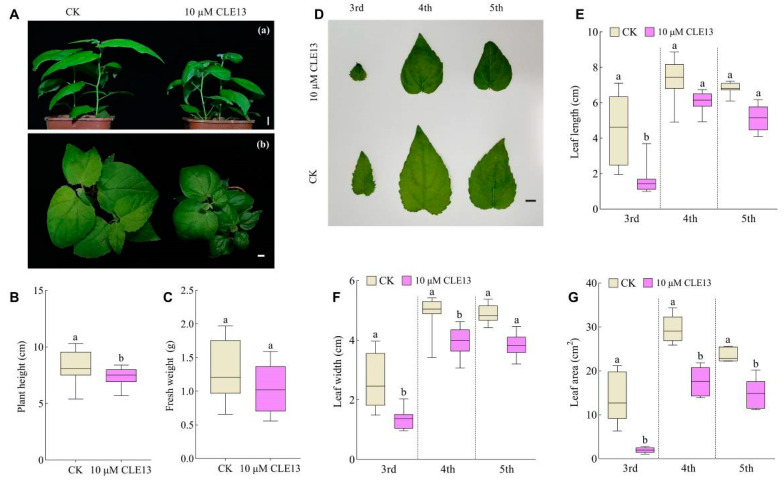
PtrCLE13 peptide suppressed the growth of *P. tomentosa* by inhibiting plant height and leaf development. (**A**) Whole plants of *P. tomentosa* untreated (control check, CK) and treated by 10 µM PtrCLE13 [(**a**) front view; (**b**) top view]. Bar = 1 cm. (**B**,**C**) Plant height (**B**) and fresh weight (**C**) of *P. tomentosa* seedlings in (**A**). (**D**), Leaves of *P. tomentosa* untreated (control check, CK) and treated by 10 µM PtrCLE13. 3rd, 4th and 5th refer to the leaves on the third, fourth and fifth nodes of *P. tomentosa* seedlings, respectively. Bar = 1 cm. (**E**–**G**) Leaf length (**E**), leaf width (**F**) and leaf area (**G**) of *P. tomentosa* seedlings in (**A**). Data are presented as means ± SD (*n* = 9 for **B**,**C**,**E**–**G**). Different lowercase letters indicate a significant difference at *p* < 0.05 based on ANOVA.

On one hand, when the *P. tomentosa* seedlings were treated with PtrCLE13 and CLE9 peptides, they developed adventitious roots in a considerably slower manner and resulted in shorter adventitious roots. Specifically, the application of 0.1 μM and 1 μM of PtrCLE13 peptide reduced the length of adventitious roots of *P. tomentosa* by 53.53% and 71.56%, respectively, compared with the control group. Additionally, the application of 0.1 μM and 1 μM of CLE9 peptide decreased the length of adventitious roots of *P. tomentosa* by 69.46% and 81.74%, respectively, relative to the control group (Figure 6A–D). To investigate the potential regulatory mechanisms underlying the inhibitory effects of PtrCLE13 and CLE9 peptides on adventitious root growth, we conducted the analysis of gene expression levels in *P. tomentosa* and *A. thaliana* roots treated with PtrCLE13 and CLE9 peptides. Compared with the control check (CK), in *Arabidopsis* roots treated with PtrCLE13 peptide, *AtARF7*, which is a key transcription factor regulating the expression of auxin response genes, showed a reduced expression; expression of transcription factors *AtLBD16* and *AtLBD29*, both involved in plant root development, was strongly suppressed (Figure 6E). Similarly, the expression levels of *AtARF5*, *AtLBD16* and *AtLBD29* in *Arabidopsis* roots treated with CLE9 peptide exhibited a significant reduction compared with the CK (Figure 6F). Additionally, in *P. tomentosa* roots treated with PtrCLE13 and CLE9 peptides, *PtoARF5.2*, which is homologous with *AtARF7*, displayed substantially lower expression (Figure 6G); *PtoLBD16.2* and *PtoLBD19*, which are homologous with *AtLBD16* and *AtLBD29*, respectively, had much lower expression (Figure 6H). Moreover, as the concentration of the peptides applied in vitro increased, the gene-downregulating effect became more prominent. These findings suggest that exogenously applied synthetic PtrCLE13 and CLE9 peptides may inhibit root growth in *Arabidopsis* and *poplar* by suppressing the expression of *ARFs* and *LBDs*. On the other hand, the application of PtrCLE13 peptide on the stem tips of *P. tomentosa* seedlings resulted in a significant inhibition of the aboveground parts of *P. tomentosa* seedlings (Figure 7A). Compared with the CK, the *P. tomentosa* treated with PtrCLE13 peptide exhibited reduced aboveground plant height and fresh weight. In particular, the plant height decreased by 10.25% compared with that of the CK (Figure 7B,C). Moreover, the width, length and area of leaves on the third, fourth and fifth nodes of *P. tomentosa* treated with PtrCLE13 peptide were reduced compared to the CK. Notably, compared to the CK, the areas of leaves on the third, fourth and fifth nodes of *P. tomentosa* treated with PtrCLE13 peptide were reduced by 85.94%, 40.56% and 38.40%, respectively (Figure 7D–G).

### 2.9. Overexpression of PtrCLE13A Enhances Osmotic and Drought Tolerance in P. tomentosa

To investigate the role of PtrCLE13 peptide in regulating drought resistance in poplar, we conducted an overexpression study of *PtrCLE13A* gene in *P. tomentosa*, considering its active response to drought stress (Figure 4D and Supplementary Figure S8A). A total of four positive lines were identified, with the expression levels of *PtrCLE13A* in OE-6 (overexpression line 6) and OE-3 (overexpression line 3) ranking highest (Supplementary Figure S10). Therefore, two transgenic lines, OE-6 and OE-3, were selected for follow-up investigation.

**Figure 8:**
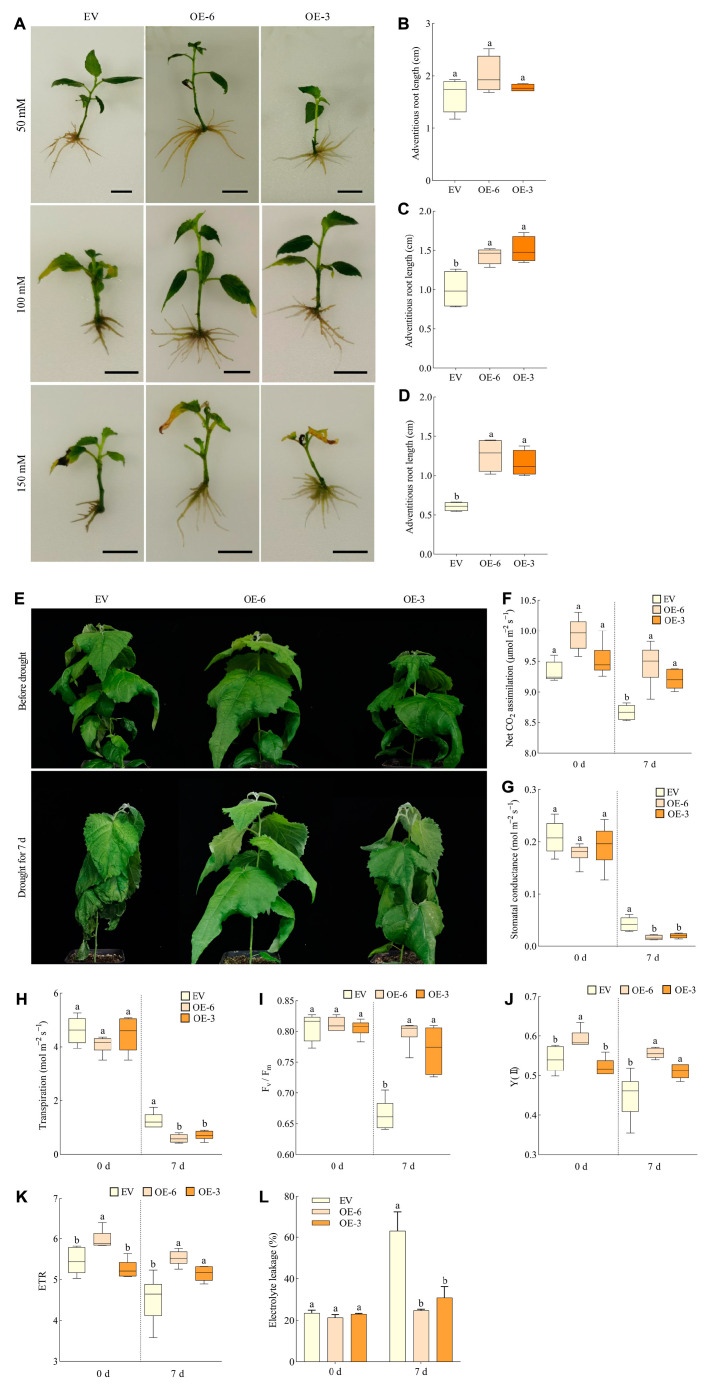
Overexpression of *PtrCLE13A* enhances osmotic and drought tolerance in *P. tomentosa.* (**A**) Morphological phenotypes of EV (empty vector), OE-6 (overexpression line 6) and OE-3 (overexpression line 3) poplar roots subjected to mannitol treatments at concentrations of 50 mM, 100 mM and 150 mM. Bar = 1 cm. (**B**–**D**) The length of adventitious roots of poplar seedlings in (**A**). Data are presented as means ± SD (*n* = 12 for (**B**–**D**)). (**E**) Phenotypes of EV, OE-6 and OE-3 poplar seedlings before and after drought stress. (**F**–**L**) Net photosynthetic rate (**F**), stomatal conductance (**G**), transpiration (**H**), Fv/Fm (**I**), Y (Ⅱ) (**J**), ETR (**K**) and relative electrolyte leakage (**L**) of leaves of poplar seedlings in (**E**). Data are presented as means ± SD (*n* = 6 for (**F**–**L**)). Different lowercase letters indicate a significant difference at *p* < 0.05 based on ANOVA.

To explore whether the overexpression of the *PtrCLE13A* gene in *P. tomentosa* enhances its tolerance to osmotic stress, we cultured EV (empty vector), OE-6 and OE-3 poplar plants on WPM rooting agar medium supplemented with 50 mM, 100 mM and 150 mM mannitol for three weeks. It was observed that the root systems of OE-6 and OE-3 poplar seedlings were more developed compared with EV when subjected to different concentrations of mannitol, particularly at 100 mM and 150 mM mannitol (Figure 8A). Furthermore, when treated with 100 mM mannitol, the adventitious roots of OE-6 and OE-3 were significantly longer than those of EV by 31.20% and 34.53%, respectively, and when treated with 150 mM mannitol, the adventitious roots of OE-6 and OE-3 were significantly longer than those of EV by 52.04% and 47.42%, respectively (Figure 8C,D). The overexpression of *PtrCLE13A* in *P. tomentosa* markedly increased adventitious root length compared with EV under the treatment of mannitol, indicating that upregulation of *PtrCLE13A* could enhance the tolerance to osmotic stress in poplar.

To further verify the differences in drought tolerance between transgenic poplars and EV, we conducted the drought treatment by withholding watering in the greenhouse. After 7-day drought treatment, the leaves of transgenic poplar seedlings displayed less severe wilting than EV (Figure 8E). There were no significant differences in net photosynthetic rate, stomatal conductance and transpiration rate among EV, OE-6 and OE-3 poplars before drought treatment. However, after drought treatment, the net photosynthetic rate of OE-6 and OE-3 poplars was significantly 8.29% and 5.85% higher than that of the EV. Moreover, the stomatal conductance of OE-6 and OE-3 was 61.05% and 52.80% lower than that of EV, respectively, and the transpiration rates of OE-6 and OE-3 were 53.24% and 44.04% lower than that of EV, respectively (Figure 8F–H). There was also no difference in Fv/Fm among EV, OE-6 and OE-3 poplars under normal growth conditions, while the Fv/Fm of EV decreased to 0.665 after drought treatment, which was remarkably lower than that of OE-6 and OE-3 poplars (Figure 8I). Before drought treatment, the Y (Ⅱ) and ETR values of OE-6 poplar leaves were significantly 8.80% and 8.82% higher than those of the EV, respectively. Under drought conditions, the Y (Ⅱ) values of the leaves of OE-6 and OE-3 poplars were significantly 19.17% and 11.97% higher than those of EV, respectively, and the ETR values of OE-6 and OE-3 were also significantly 17.80% and 11.97% higher than those of EV, respectively (Figure 8J,K). In addition, electrolyte leakage was also determined to assess plasma membrane damage among EV, OE-6 and OE-3 poplars. Under drought stress, the electrolyte leakage rate of OE-6 and OE-3 poplar leaves suffered a significantly lower magnitude compared with that of EV (Figure 8L), suggesting reduced membrane damage in transgenic plants relative to EV plants. Conclusively, these results indicate that poplars overexpressing *PtrCLE13A* exhibit better photosynthetic performance and less cell membrane damage under drought conditions, demonstrating that *PtrCLE13A* can positively enhance the drought tolerance of poplars.

## 3. Discussion

Previous studies have already conducted genome-wide identifications of the CLE gene family in plants such as *A. thaliana*, *Oryza sativa*, *Zea mays*, *Triticum aestivum*, *Glycine max*, *Phaseolus vulgaris*, *Gossypium hirsutum*, *Gossypium raimondii*, *Gossypium arboreum*, *Brassica napus*, *Brassica rapa*, *Brassica oleracea*, *Vitis vinifera*, *Cucumis sativus*, *Raphanus sativus*, *Pinophyta* and *P. trichocarpa* [23,24,25,26,27,28,29,30,31,32,33,34]. Given that there has been very little work on the identification of the *CLE* gene family in woody plants, this study carried out genome-wide identifications of the *CLE* gene family in *P. tomentosa* and *P. alba* × *P. glandulosa* for the first time and also made a relatively in-depth comparative analysis of them with the *CLEs* of *P. trichocarpa*.

The results of this study show that *P. trichocarpa*, *P. tomentosa* and *P. alba* × *P. glandulosa* contain 52, 45 and 89 *CLE* genes, respectively. All three poplar species are diploids, but the number of *CLE* genes in *P. alba* × *P. glandulosa* is 1.71 times and 1.98 times that in *P. trichocarpa* and *P. tomentosa*, respectively. Studies on *CLE* genes in other plants of the same genus have shown that plants in the genus *Brassica*, specifically, *B. napus* (4×), *B. rapa* (2×) and *B. oleracea* (2×), have 70, 29 and 32 *CLE* genes, respectively. Moreover, plants in the genus *Gossypium*, namely, *G. raimondii* (2×), *G. arboreum* (2×) and *G. hirsutum* (4×), have 55, 56 and 86 *CLE* genes. The differences in the number of *CLE* genes among these plants of the same genus are related to their ploidy levels, but this is not the case for the three poplar species in our study [24,25,34]. Genome evolution analysis shows that approximately five million years ago, *P. alba* and *P. trichocarpa* underwent species differentiation. *P. alba* gave rise to an independent variety, *P. bolleana*, around 4.8 million years ago [35]. Later, *P. bolleana* (♂) and *P. adenopoda* (♀) hybridized to produce *P. tomentosa* around 3.93 million years ago [36]. In modern times, *P. alba* (♀) and *P. glandulosa* (♂) have been artificially hybridized to produce *P. alba* × *P. glandulosa* [37]. In one respect, *P. alba* × *P. glandulosa* is a modern hybrid variety, which selected parents with fast growth and strong resistance; some genes in *P. alba* and *P. glandulosa* have expanded through selective pressure, and the gene number after hybridization may be higher than that in other poplar varieties and has been preserved [38,39]. In another respect, during the long-term evolution, in the *CLE* gene families of *P. trichocarpa* and *P. tomentosa*, some genes may be dispensable for environmental adaptation or may become more adaptable to the environment after mutation, and due to the accumulation of harmful gene mutations, chromosomal structural variations and other reasons, the number of *CLE* genes in these two species of poplar has decreased [40].

The number of *CLE* genes in each type of poplar does not have a one-to-one correspondence with the number of mature peptides that are ultimately formed. Instead, the mature peptides formed from the precursor proteins of one to multiple *CLE* genes are often exactly the same, which increases the functional redundancy of *CLE* genes.

The A sub-genome and D sub-genome of *P. tomentosa*, as well as the A sub-genome and G sub-genome of *P. alba* × *P. glandulosa*, are two groups of homologous sub-genomes. Therefore, the *CLE* genes of these two types of poplars are considered to be double genes. However, there are also cases where corresponding homologous genes are missing on the chromosomes, which may be due to the loss of these homologous genes during the evolutionary process or becoming pseudogenes as a result of insertion, duplication or deletion events [41].

This study found that all the duplication patterns of collinear gene pairs within or between the three poplar species are WGD or segmental duplication. Therefore, WGD or segmental duplication is the main driving force for the expansion of the *CLE* gene family in the three poplar species during the evolutionary process. This is similar to the amplification pattern of the *CLE* gene family in cotton but different from the tandem duplication pattern of the *CLE* gene family in wheat, indicating that there are differences in the amplification patterns of the same gene family in different species [24,29].

The molecular weights of the precursor proteins encoded by the *CLE* genes in *A. thaliana* are all < 15 kDa. However, three, ten and two CLE precursor proteins with molecular weights > 15 kDa are found in *P. trichocarpa*, *P. tomentosa* and *P. alba* × *P. glandulosa*, respectively. Moreover, some of the precursor proteins are even > 20 kDa. This may be related to the C-terminal extension of the protein. The C-terminal extension of the protein is of great significance for the function, stability and activity of the protein [42,43].

This study found that most of the *CLE* genes in *P. tomentosa* have introns and have very few UTR regions, which is different from the situation in the other two poplar species. During the evolutionary process, it is likely that due to different selective pressures, the *CLE* genes in *P. tomentosa* have retained the intron structure. After all, the presence of introns can increase the complexity of gene expression regulation. For example, introns can interact with transcription factors and promote gene expression, or the enhancers within introns can recruit transcriptional activators, which facilitate the binding of RNA polymerase II to the promoter, thereby initiating gene transcription [44]. In contrast, the other two poplar species may have undergone the simplification of gene structures during evolution, achieving a more efficient gene expression pattern by losing introns [45]. Furthermore, the pre-mRNA containing introns needs to be precisely processed by the spliceosome to form mature mRNA. In *P. tomentosa*, this splicing process may be a meticulous regulatory step to ensure the correct expression of CLE proteins. However, due to the differences in gene structures, the other two poplar species may adopt different post-transcriptional processing strategies, such as a more direct mRNA processing method that does not require a complex intron splicing process. As for the rarity of UTR in the *CLE* gene structure of *P. tomentosa*, it may be caused by factors such as chromosomal structural variations, abnormal transcription initiation, premature transcription termination and abnormal mRNA processing [46,47].

The expression patterns of genes reflect, to a certain extent, the spatiotemporal characteristics of how genes exert their functions. Under normal growth conditions, the expression levels of *PtrCLE13B* and *PtrCLE13C* are relatively high in the roots of poplar trees, while this is not the case for *PtrCLE13A*. This study found that the mature peptides encoded by *PtrCLE13A*/*B*/*C* are completely identical. Exogenous addition of this peptide inhibited the development of poplar roots. It is speculated that the low expression level of *PtrCLE13*A in poplar roots may be to maintain the homeostasis of the total amount of PtrCLE13 peptides so as to maintain the normal development of poplar roots, and this needs further verification. In addition, the expression levels of *PtrCLE13A*/*B*/*C* are all high in the xylem of poplar trees, which is consistent with previous research results [20]. This implies that PtrCLE13 peptides may play a role in the development of poplar xylem, and this also requires further verification. The results of subcellular localization of preCLE13A protein indicate that it is located on the cell membrane, which is similar to the subcellular localization results of PAMP-INDUCED SECRETED PEPTIDE 3 (PIP3) and EPIDERMAL PATTERNING FACTOR-LIKE 6 (EPFL6), also secreted peptides [48,49]. This may be adapted to its function.

In vitro application experiments of *Arabidopsis* CLE mature peptides at a certain concentration showed that, except for AtCLE42, AtCLE46 and AtCLE1/3/4, other peptides had a significant inhibitory effect on the elongation of roots in *Arabidopsis* [22]. Similarly, this study found that PtrCLE13 and AtCLE9 mature peptides at a certain concentration also had a significant inhibitory effect on the elongation of *Arabidopsis* and poplar roots. Although there are 1–2 amino acid differences among the mature peptides of PtrCLE13 (RLVPTGPNPLHH), AtCLE9 (RLVPSGPNPLHN) and AtCLE13 (RLVPSGPNPLHH), they have similar functions. Previous studies have shown that the transcriptional activation of *LBD16* and *LBD29* by TDIF can be attributed to two aspects. On the one hand, the TDIF-TDR-BIN2 module can activate ARF7 and ARF19 through the phosphorylation pathway, weakening their interaction with the AUX/IAA repressor complex, thus enhancing their transcriptional activation of *LBD16* and *LBD29* [14]. On the other hand, the overexpression of *TDIF* enhances the de novo transcription of *ARF7* and *ARF19* [50]. Therefore, in the roots of *A. thaliana* treated with PtrCLE13 and AtCLE9 peptides, the de novo transcription of *AtARF7* is decreased, which in turn weakens the expression levels of *AtLBD16* and *AtLBD19*, resulting in the inhibition of root growth in *A. thaliana*. In addition, *LBD16* is involved in the transformation of cell fate, which is crucial for the initiation of adventitious roots from non-root tissues of plants, such as stems and leaves, and this study found that the AUX1/LAX3-ARF7/ARF19-LBD16/LBD18 signaling module is also crucial for the formation of adventitious roots in *A. thaliana* [51,52,53]. Similarly, in the adventitious roots of poplar generated under the treatment of PtrCLE13 and AtCLE9 peptides, the reduced de novo transcription of *PtoARF5.2* may lead to decreased expression levels of *PtoLBD16.2* and *PtoLBD29*, thereby inhibiting the development of adventitious roots in poplar, and this requires further research to prove. Moreover, the amino acid sequences of AtCLE9, PtrCLE9 and PtrCLE10A/B mature peptides are completely identical, and this implies that the functions and mechanisms of action of PtrCLE9/10A/B mature peptides are likely to be similar to those of AtCLE9.

In higher plants, specific cells in the peripheral region of the shoot apical meristem begin to differentiate and form leaf primordia under the induction of a series of signals, such as changes in the concentration gradient of plant hormones and regulation of gene expression. For example, through the polar transport of auxin, a local area with a high concentration of auxin is formed around the SAM of plants. This activates the expression of relevant genes, causing the cells to change their fate and start differentiating into leaf primordium cells [54,55]. This study found that the exogenous addition of PtrCLE13 peptide at a certain concentration reduced the plant height and leaf area of *P. tomentosa*. This might be because the signal pathway mediated by the PtrCLE13 peptide affected the concentration gradient of plant hormones and the expression of related genes in the SAM of *P. tomentosa*, resulting in the hindrance of SAM and leaf development, and this requires further research to prove.

In the face of drought stress, the stress response pathways in plants will be activated due to the plant hormone signaling, as well as the production and mobilization of antioxidants and metabolites. Subsequently, plants will actively maintain the physiological water balance by increasing the water uptake of roots from the soil, reducing water loss by closing stomata and regulating the osmotic processes within tissues [56]. The timely closure of stomata or a certain degree of reduction in stomatal density under drought stress both play a positive role in reducing water loss in plants. For example, the stomatal closure mediated by the CLE25 peptide and the reduction in stomatal density mediated by EPFL6 peptide have enhanced the drought tolerance of *A. thaliana* and poplar trees, respectively [49,57]. In this study, compared with EV, the poplar lines overexpressing *PtrCLE13A* (OE lines) showed slightly lower stomatal conductance and transpiration rate under well-watered conditions. However, under drought stress conditions, the stomatal conductance and transpiration rate of the OE lines were significantly lower than those of EV, indicating that PtrCLE13A peptide reduces the water loss of poplar by reducing stomatal conductance and transpiration rate under drought conditions. In addition, the maximal quantum yield of photosystem II (Fv/Fm) reflects the potential maximum light energy conversion efficiency of plants. Under normal conditions, this value ranges from 0.8 to 0.85 in the vast majority of higher plants [58]. However, under adverse stress conditions, this value will decrease [59]. Y (Ⅱ) represents the quantum yield of photochemical energy conversion, which reflects the actual light energy conversion efficiency of plants in photosystem II [60]. When exposed to drought stress, the membrane system will be damaged, thus affecting the electron transport efficiency of plant photosystem II. Nevertheless, the OE lines have higher Fv/Fm, Y (Ⅱ) and ETR values compared to the EV plants, which enables them to suffer less damage under drought stress. Additionally, electrolyte leakage is an indication of plasma membrane damage. Under drought stress, the electrolyte leakage rate of EV was higher than that of the OE lines, suggesting that the EV plants suffered more severe membrane damage under drought stress. This study also found that the adventitious roots of the OE lines were longer than those of the EV under osmotic stress treatment, which indicates that the OE lines have a better ability for roots to absorb water. These results and analyses all support the conclusion that the overexpression of *PtrCLE13A* enhances the drought tolerance of poplar.

In this study, the *CLE* gene families of *P. tomentosa* and *P. alba* × *P. glandulosa* were identified for the first time. Bioinformatic analysis of *CLE* genes in three poplar species has revealed the conservation, diversity and evolutionary relationship of *CLE* genes among different poplar species. In addition, our research has, to a certain extent, filled the gap in understanding the functions of A-type *CLEs* in poplar trees in terms of stress resistance. However, the specific signaling pathways mediated by PtrCLE13A and its receptor have not yet been clearly defined. In addition to its role in stress resistance, PtrCLE13A may also affect the development of poplar wood. Moreover, gene editing techniques can be used to create mutant poplar plants, which can help to further explore the cell communication processes mediated by CLE peptides and their receptors in poplar trees. Alternatively, by editing *CLE* genes or their promoter regions, new poplar varieties with good stress resistance can be developed.

## 4. Materials and Methods

### 4.1. Sequence Search and Identification of Poplar CLE Genes

To identify the members of the *CLE* gene family in *P. tomentosa* and *P. alba* × *P. glandulosa*, protein sequences from *P. trichocarpa* in the published literature were used as query sequences [20,23]. The genome files and protein sequences of *P. tomentosa* and *P. alba* × *P. glandulosa* were downloaded from the NCBI (https://www.ncbi.nlm.nih.gov/ (accessed on 20 February 2024). The BLAST search was performed using TBtools-Ⅱ version 2.095 (https://github.com/CJ-Chen/TBtools (accessed on16 March 2024) with an e-value cutoff of 1 × 10^−10^ [61]. To further determine whether these genes are *CLE* gene family members, the typical CLE functional domains were analyzed using HmmerWeb version 2.41.1 (https://www.ebi.ac.uk/Tools/hmmer/ (accessed on 17 March 2024).

### 4.2. Analysis of CLE Gene Structures and CLE Protein Features

The structure of the *CLE* genes was displayed using TBtools-Ⅱ version 2.095. The number of amino acids (AAs), molecular weight (Mw) and isoelectric point (pI) of the candidate members were calculated according to the ExPASy server (http://web.expasy.org/compute_pi/ (accessed on 20 March 2024) [62]. The CLE signal peptide cleavage sites were predicted using SignalP-6.0 (https://services.healthtech.dtu.dk/services/SignalP-6.0/ (accessed on 23 March 2024) [63].

### 4.3. Multiple Sequence Alignment Analysis and Construction of Phylogenetic Trees

ClustalX version 2.0 (EMBL Outstation-European Bioinformatics Institute, Wellcome Trust Genome Campus, Hinxton, Cambridge, UK)was used to perform multi-sequence alignment of poplar CLE domain sequences with default parameters [64], and the results were highlighted with colors using Jalview (http://www.jalview.org/ (accessed on 2 April 2024) [65]. The CLE motifs were aligned using ClustalW version 2.0, and the unrooted phylogenetic trees were constructed using MEGA version 7.0 with the neighbor-joining (NJ) method [66]. To support the presumed evolutionary relationships, the bootstrap method was used with 1000 replicates. Weblogo 3 (https://weblogo.berkeley.edu/logo.cgi (accessed on 5 April 2024) was used to predict the CLE functional domains [67].

### 4.4. Analysis of Collinearity, Duplication and Ka/Ks Values

The gene duplication events were analyzed using the Advanced Circos of TBtools-Ⅱ version 2.095. To exhibit segmentally duplicated pairs and orthologous pairs of *CLE* genes, the Multiple Synteny Plotter in TBtools was used to draw collinearity maps [68]. The Ka (non-synonymous substitution rate)/Ks (synonymous substitution rate) ratios of the *CLE* genes were calculated using KaKs_calculator version 2.0 [69].

### 4.5. Analysis of Cis-Acting Elements of Promoter Regions

The promoter sequences (2000 bp upstream of the initiation codon “ATG”) of all *CLE* gene members were filtered from three poplar genomes using TBtools-Ⅱ version 2.095. Putative cis-acting elements were identified using the online Plant CARE server (https://bioinformatics.psb.ugent.be/webtools/plantcare/html/ (accessed on 7 April 2024), and the results were visualized through the generation of heatmaps using GraphPad Prism version 8.3.0 (GraphPad Software Inc., San Diego, CA, USA).

### 4.6. Subcellular Protein Localization

For subcellular localization of the pre-propeptide of PtrCLE13A, 35S: preCLE13A-eGFP fusion protein construct and control 35S:eGFP construct were infiltrated into *N. benthamiana* leaves. At the same time, 35S: preCLE13A-eGFP and pm-ck CD3-1001 were infiltrated into tobacco leaves together. The pm-ck CD3-1001 was used as a plasma membrane marker, and the vector contains a membrane protein labeled with cyan fluorescent protein [49,70]. The tobacco plants subjected to transient infiltration were placed in the dark for 12 h, then cultivated under normal light conditions. Three days later, the injected leaves were torn with forceps to make temporary slides for observation. Fluorescence was detected at 515 ± 15 nm for GFP, 485 ± 20 nm for CFP and 660–700 nm for chlorophyll. An argon ion laser and a 405 nm PIN diode laser were used in the experiment. Images were captured by confocal fluorescence microscopy (SP8, Leica, Wetzlar, Germany).

### 4.7. P. trichocarpa Growth Conditions and Dehydration Treatment

Three-month-old seedlings of *P. trichocarpa* were grown individually in pots containing a mixture of soil and vermiculite (2:1) at 22 °C under a 16 h/8 h (light/dark) photoperiod. For the dehydration treatment, three-month-old seedlings were removed from the soil, and the roots were exposed to air at 50% relative humidity and 25 °C under dim light for 6 h. Then, leaves were collected from the third and fifth internodes at different time points (0 h, 2 h, 4 h and 6 h) and frozen immediately in liquid nitrogen. We simultaneously collected the following tissues from the three-month-old *P. trichocarpa* plants: root, young stem, bark, xylem, young leaf and mature leaf.

### 4.8. Synthetic CLE Peptides

The PtrCLE13 (RLVP^Hyp^TGP^Hyp^NPLHH) and CLE9 (RLVP^Hyp^SGP^Hyp^NPLHN) peptides were synthesized from Sangon Biotech (Shanghai, China). The purity of these peptides is at least 95%, and they exhibited solubility in H_2_O.

### 4.9. Synthetic PtrCLE13 and CLE9 Peptide Treatment

All *Arabidopsis* genotypes used in this study are the Columbia (*Col-0*) ecotype. The seeds were sterilized with 75% ethanol solution for 30 s and 1% sodium hypochlorite for 5 min, rinsed five times with H_2_O and stratified at 4 °C for 2 days before the peptide treatment. For the peptide treatment, the seeds of the control check (CK) were grown in Petri dishes containing sterilized half-strength Murashige and Skoog (½ MS) liquid medium with 0.5% (*w*/*v*) sucrose (pH = 5.7), while the seeds of the treatment groups were grown in the same medium plus 0.1, 0.5, 1.0, 5.0, 10.0 and 20.0 µM of PtrCLE13 or CLE9 peptides. Three independent experimental trials were conducted with at least 15 seeds for each treatment in each trial. The Petri dishes were transferred to a growth room maintained at 22 °C under a 16/8 h (light/dark) photoperiod for 12 days, then the seedlings were photographed, and the length of primary roots was measured using ImageJ version 1.54. At the same time, the root tip was observed under stereo-fluorescence microscope (M205FA, Leica, Germany), and the lengths of the meristematic zone (MZ) and elongation zone (EZ) were measured using ImageJ. The treated root tissues of *A. thaliana* seedlings were collected and quickly frozen with liquid nitrogen.

For *P. tomentosa* seedling treatment, on the one hand, wild-type young shoots with equal numbers of leaves and internodes that had identical growth cycles were grown in bottles containing solid wood plant medium (WPM) with 0, 0.1 and 1 µM of PtrCLE13 or CLE9 peptides under a phytotron with a 16/8 h (light/dark) cycle at 22 °C. Three independent experimental trials were conducted with at least 10 seedlings for each treatment in each trial. After three weeks, the adventitious root length of the poplar seedlings was measured. The treated root tissues of *P. tomentosa* seedlings were collected and quickly frozen with liquid nitrogen. On the other hand, the one-month-old *P. tomentosa* micro-propagated seedlings with the same size and growth cycles were transferred to pots containing a mixture of soil and vermiculite (2:1) at 22 °C under a 16 h/8 h (light/dark) photoperiod (150 μmol m^−2^ s ^−1^) and 70% relative humidity for growth. After 2 days, 100 µL of 10 µM PtrCLE13 peptide was added to the stem tips of poplar seedlings in the treatment group once a day by a micropipette, while an equal amount of solvent H_2_O was applied to the stem tips of poplar seedlings in the control check. Three independent experimental trials were conducted with at least 5 seedlings for each treatment in each trial. After three weeks of peptide treatment, plant height, fresh weight, leaf width, leaf length and leaf area of poplar seedlings were measured.

### 4.10. RNA Extraction and RT-qPCR Analysis

Total RNA was extracted from collected materials by using an EASYspin Plus Plant RNA Kit (Aidlab Bio Inc., Beijing, China). A NanoDrop 2000 Spectrophotometer (Thermo, West Palm Beach Inc., West Palm Beach, FL, USA) was used to assess the quantity and quality of RNA. Then, Quant One Step RT-PCR kit (Tiangen Bio Inc., Beijing, China) was used for reverse transcription following the manufacturer’s protocol. Subsequently, cDNA was used as the template for RT-qPCR quantification. RT-qPCR was performed using the CFX Connect™ Real-Time PCR System (BIO-RAD Inc., Hercules, CA, USA) in accordance with the manufacturer’s instructions. The reaction mixture for the RT-qPCR analysis comprised 1 μL (∼100 ng) template, 0.5 μL (0.2 μM) forward primer, 0.5 μL (0.2 μM) reverse primer, 10.5 μL RNase-free ddH_2_O and 12.5 μL 2 × SYBR qPCR Mix in a total volume of 25 μL. The RT-qPCR program consisted of an initial temperature of 94 °C for 2 min, followed by 40 cycles of 94 °C for 10 s and 58 °C for 30 s. A melting curve was constructed by increasing the temperature from 68 to 99 °C at a rate of 0.05 °C s^−1^. *PtActin* and *PtUBQ* as well as *AtActin2* and *AtPP2A* served as double internal reference genes in poplar and *A. thaliana*, respectively. The ratio = (*E*_t_)^Δ*CT*t^/*NF* method was used to calculate the relative expression value [71]. Each experiment was based on three biological replicates of each sample and three technical replicates of each biological replicate. Three independent experimental trials were conducted with at least 3 seedlings in each trial. All primers used are listed in Supplementary Table S11.

### 4.11. Plasmid Construction and Genetic Transformation

The cDNA sequence of *preCLE13A* was amplified by PCR from the total cDNA of *P. trichocarpa* and cloned into the modified pCAMBIA-1301 vector driven by the CaMV35S promoter (Supplementary Figure S9). The constructed vector and empty vector were transferred into *A. tumefaciens* strain EHA105 and transformed into wild-type triploid white poplar (WT) by the leaf disc method [72]. In brief, one- to two-month-old aseptic poplar seedlings were used for genetic transformation. The poplar leaves were cut into small pieces and dipped into the infection medium, which contained the *A. tumefaciens* incubated in YEB medium overnight till the OD_600_ = 0.4–0.6 and 100 μM acetosyringone, for around 8–15 min with gentle shaking. The infected leaves were incubated on differentiation medium [WPM containing 1.0 mg L^−1^ naphthalene acetic acid (NAA), 2.0 mg L^−1^ zeatin (ZT), 400 mg L^−1^ cefotaxime, 100 μmol L^−1^ acetosyringone, 30 g L^−1^ sucrose and 0.6% (*w*/*v*) agar] and carried out in the dark for two days. Then, leaves were transferred to selected medium [WPM containing 1.0 mg L^−1^ NAA, 2.0 mg L^−1^ ZT, 400 mg L^−1^ cefotaxime, 9 mg L^−1^ hygromycin, 30 g L^−1^ sucrose and 0.6% (*w*/*v*) agar] for callus induction in the light. The formed calluses were transferred to the budding medium [WPM containing 0.1 mg L^−1^ NAA, 2.0 mg L^−1^ ZT, 400 mg L^−1^ cefotaxime, 9 mg L^−1^ hygromycin, 30 g L^−1^ sucrose and 0.6% (*w*/*v*) agar] for bud induction. Finally, the regenerated buds were transferred to selected rooting medium [WPM containing 0.1 mg L^−1^ NAA, 400 mg L^−1^ cefotaxime, 9 mg L^−1^ hygromycin, 30 g L^−1^ sucrose and 0.6% (*w*/*v*) agar]. The regenerated poplar seedlings with established root systems were utilized for transgenic identification.

### 4.12. Molecular Verification and Histochemical Staining of Transgenic Plants

The cetyltrimethylammonium bromide (CTAB) method was used to extract genomic DNA from transformed empty vector (EV) triploid white poplars and transgenic overexpressed (OE) lines [73]. Transformants were identified through PCR using the combination of a forward primer for *PtrCLE13A* and a reverse primer for the vector (Supplementary Table S11). The transcript levels of *PtrCLE13A* in transgenic poplars (OE-2, OE-3, OE-4 and OE-6) were confirmed using RT-qPCR. Total RNA was extracted from leaves of EV and OE poplars. cDNA synthesis was performed with a Quant One Step RT-PCR kit according to the manufacturer’s instructions. *Actin* and *UBQ* were used as the internal control [74]. GUS (β-glucuronidase) activity detection was performed by histochemical staining. Fresh poplar leaves from WT, EV and OE poplars were submerged in GUS reaction buffer and incubated at 37 °C for 12 h. Stained samples were discolored using 75 % alcohol 3–4 times and photographed.

### 4.13. Assessment of Drought Tolerance

We used mannitol to simulate osmotic stress. The young shoots from the same part of EV and OE (OE-6 and OE-3) poplars, each with an equal number of leaves and internodes, and exhibiting identical growth cycles, were cultured in WPM solid medium containing 50 mM, 100 mM and 150 mM mannitol for a duration of four weeks. Three independent experimental trials were conducted with at least 6 shoots for each treatment in each trial. Finally, the adventitious root length of them was measured. For the drought experiment, six-week-old poplar seedlings of EV and OE (OE-6 and OE-3) in the same pots (150 cm width and 135 cm height) containing identical soil conditions were subjected to drought by withholding watering for seven days in a greenhouse (temperature, 20–24 °C; light cycle: 16 h light/8 h dark period). Three independent experimental trials were conducted with at least 3 poplar seedlings for each treatment in each trial.

### 4.14. Physiological Analyses

The net CO_2_ assimilation, stomatal conduction and transpiration of the 6th to 8th leaves of EV, OE-6 and OE-3 poplars were measured by a Li-6400 Portable Photosynthesis System (Li-Cor Inc., Lincoln, NE, USA) before and after drought. The photosynthetically active radiation during the measurement was 800 μmol m^−2^ s^−1^ with 500 μmol mol^−1^ external CO_2_. The Fv/Fm, Y (Ⅱ) and ETR of the 6th to 8th leaves of EV, OE-6 and OE-3 poplars were measured using a Dual-PAM-100 measuring system (Walz Heinz GmbH, Effeltrich, Germany) before and after drought. All poplars were dark-adapted for 30 min before measurement. For measurement of leaf electrolyte leakage, on the 0th and 7th day of drought treatment, leaves were sampled, and their electrical conductivity was measured by a DDS-307 Conductivity Meter (Leici, Shanghai, China). Each measurement experiment was based on at least three biological replicates of each sample and three technical replicates of each biological replicate.

### 4.15. Statistical Analysis

All data were subjected to an analysis of variance by SPSS version 21 (SPSS Inc., Chicago, IL, USA). Values are presented as mean ± standard error (SE). The Tukey and Duncan multiple range tests were used to detect the significant differences between individual means.

## Data Availability

Data is contained within the article and Supplementary Materials.

## Supplementary Materials

The supporting information can be downloaded at: https://www.mdpi.com/article/10.3390/ijms26051944/s1.
