# Supplementary material for: Genome-Wide Identification and Functional Analysis of CLAVATA3/EMBRYO SURROUNDING REGION-RELATED (CLE) in Three Populus Species
Source: Int J Mol Sci. 2025 Feb 24;26(5):1944. doi: 10.3390/ijms26051944 (PMC11900962; doi:10.3390/ijms26051944)
Supplement: Supplementary file 1 [file ijms-26-01944-s001.zip › Supplementary Figures.pdf]

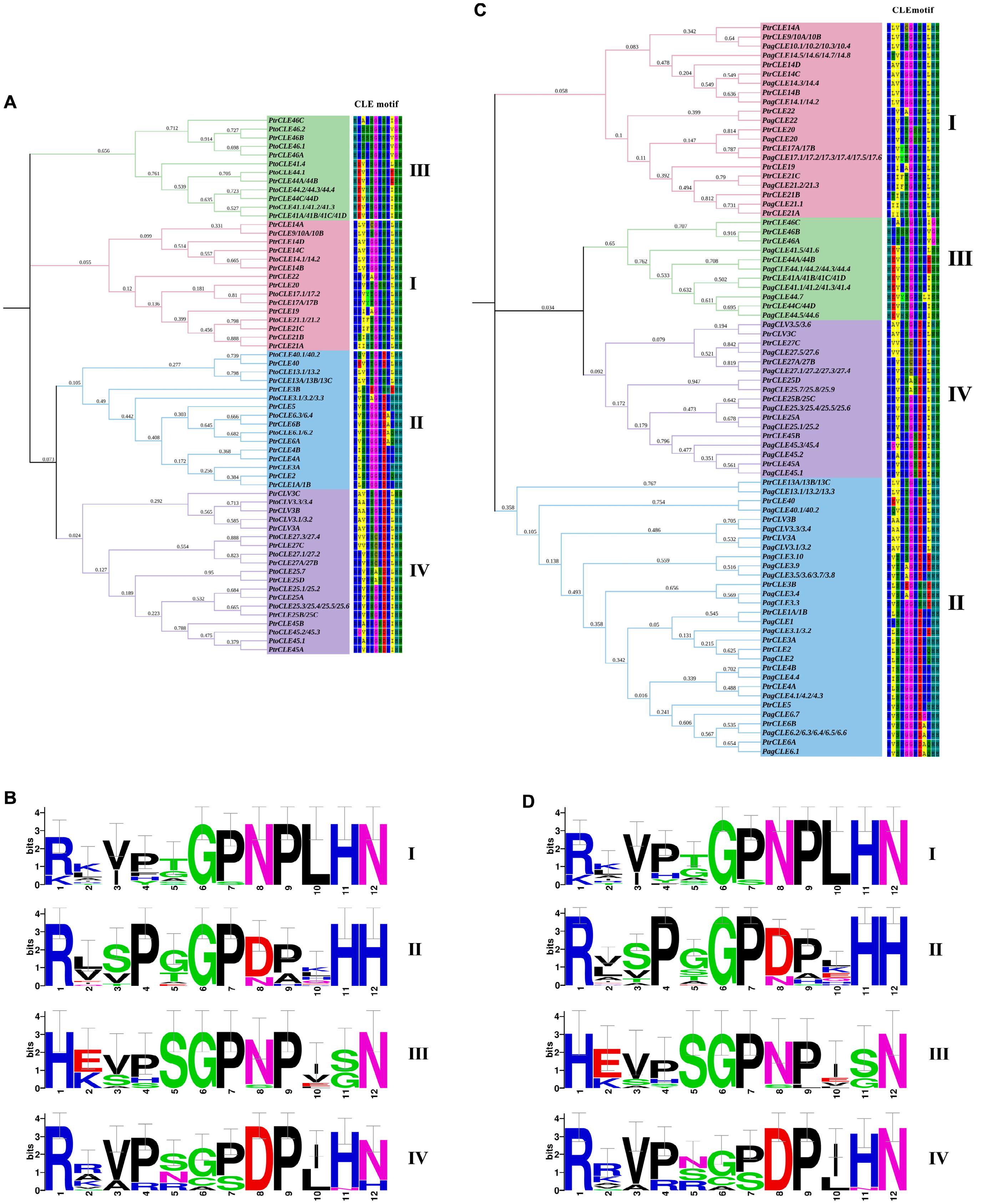

Supplementary Figure S1. Phylogenetic trees were established using MEGA7 with the NJ method. A, Phylogenetic tree of *CLEs* between *P. trichocarpa* and *P. tomentosa*. B, The weblogo represents CLE motifs (12 conserved amino acids) of four subfamilies of *P. trichocarpa* and *P. tomentosa*. C, Phylogenetic tree of *CLEs* between *P. trichocarpa* and *P. alba* × *P. glandulosa*. D, The weblogo represents CLE motifs (12 conserved amino acids) of four subfamilies of *P. trichocarpa* and *P. alba* × *P. glandulosa*.

.

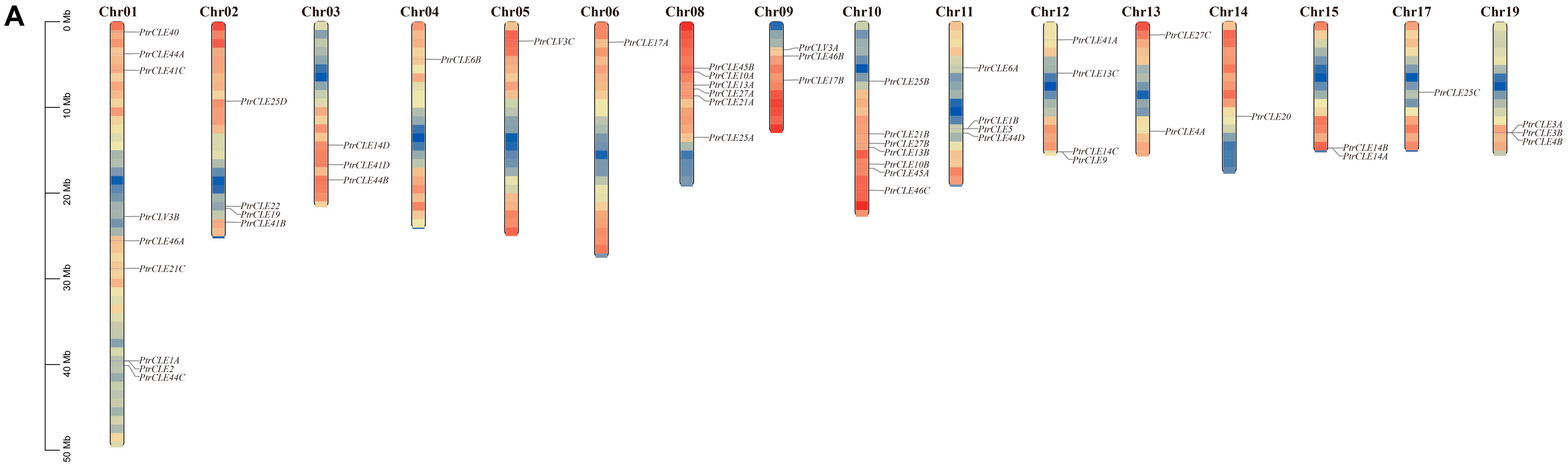
A
0 Mb
10 Mb
20 Mb
30 Mb
40 Mb
50 Mb
Chr01
PtrCLE40
PtrCLE44A
PtrCLE41C
PtrCLV3B
PtrCLE46A
PtrCLE21C
PtrCLE1A
PtrCLE2
PtrCLE44C
Chr02
PtrCLE25D
PtrCLE22
PtrCLE19
PtrCLE41B
Chr03
PtrCLE14D
PtrCLE41D
PtrCLE44B
Chr04
PtrCLE6B
Chr05
PtrCLV3C
Chr06
PtrCLE17A
Chr08
PtrCLE45B
PtrCLE10A
PtrCLE13A
PtrCLE27A
PtrCLE21A
PtrCLE25A
Chr09
PtrCLV3A
PtrCLE46B
PtrCLE17B
Chr10
PtrCLE25B
PtrCLE21B
PtrCLE27B
PtrCLE13B
PtrCLE10B
PtrCLE45A
PtrCLE46C
Chr11
PtrCLE6A
PtrCLE1B
PtrCLE5
PtrCLE44D
Chr12
PtrCLE41A
PtrCLE13C
PtrCLE14C
PtrCLE9
Chr13
PtrCLE27C
PtrCLE4A
Chr14
PtrCLE20
Chr15
PtrCLE14B
PtrCLE14A
Chr17
PtrCLE25C
Chr19
PtrCLE3A
PtrCLE3B
PtrCLE4B

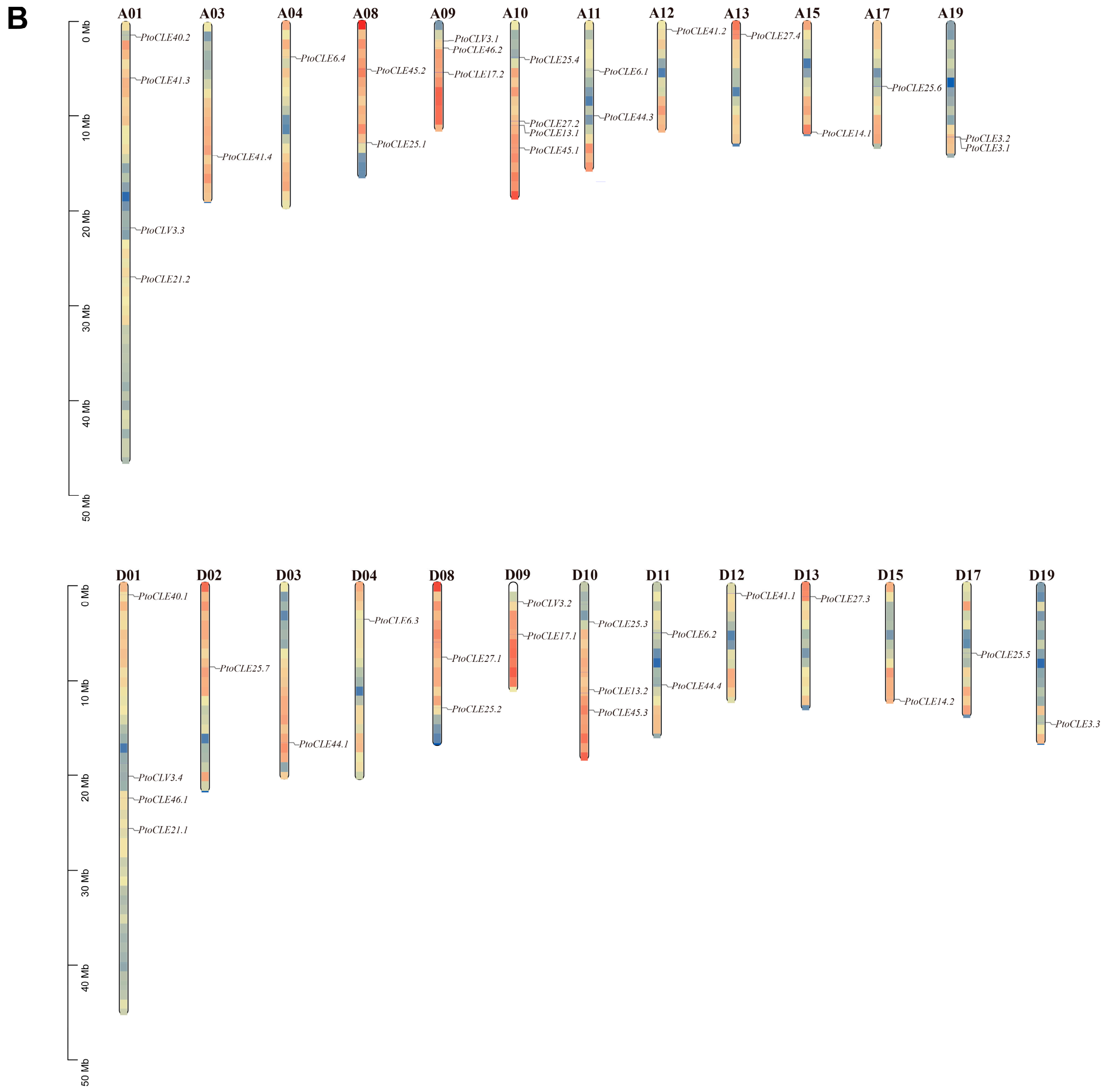
B
A01
A03
A04
A08
A09
A10
A11
A12
A13
A15
A17
A19
0 Mb
10 Mb
20 Mb
30 Mb
40 Mb
50 Mb
PtoCLE40.2
PtoCLE41.3
PtoCLV3.3
PtoCLE21.2
PtoCLE41.4
PtoCLE6.4
PtoCLE45.2
PtoCLE25.1
PtoCLV3.1
PtoCLE46.2
PtoCLE17.2
PtoCLE25.4
PtoCLE27.2
PtoCLE13.1
PtoCLE45.1
PtoCLE6.1
PtoCLE44.3
PtoCLE41.2
PtoCLE27.4
PtoCLE14.1
PtoCLE25.6
PtoCLE3.2
PtoCLE3.1
D01
D02
D03
D04
D08
D09
D10
D11
D12
D13
D15
D17
D19
PtoCLE40.1
PtoCLV3.4
PtoCLE46.1
PtoCLE21.1
PtoCLE25.7
PtoCLE44.1
PtoCLE6.3
PtoCLE27.1
PtoCLE25.2
PtoCLV3.2
PtoCLE17.1
PtoCLE25.3
PtoCLE13.2
PtoCLE45.3
PtoCLE6.2
PtoCLE44.4
PtoCLE41.1
PtoCLE27.3
PtoCLE14.2
PtoCLE25.5
PtoCLE3.3

C

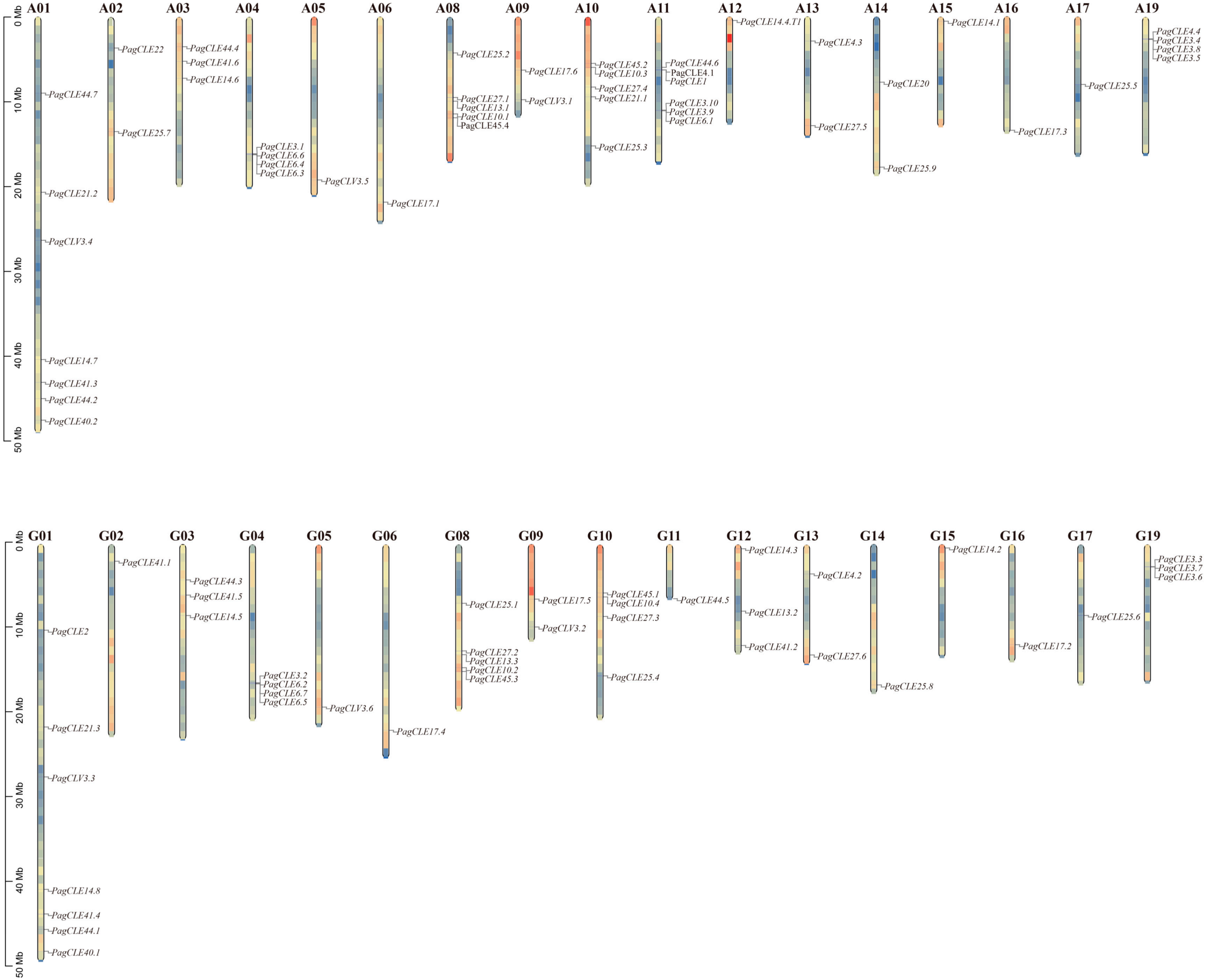

A01
A02
A03
A04
A05
A06
A08
A09
A10
A11
A12
A13
A14
A15
A16
A17
A19
0 Mb
10 Mb
20 Mb
30 Mb
40 Mb
50 Mb
PagCLE44.7
PagCLE21.2
PagCLV3.4
PagCLE14.7
PagCLE41.3
PagCLE44.2
PagCLE40.2
PagCLE22
PagCLE25.7
PagCLE44.4
PagCLE41.6
PagCLE14.6
PagCLE3.1
PagCLE6.6
PagCLE6.4
PagCLE6.3
PagCLV3.5
PagCLE17.1
PagCLE25.2
PagCLE27.1
PagCLE13.1
PagCLE10.1
PagCLE45.4
PagCLE17.6
PagCLV3.1
PagCLE45.2
PagCLE10.3
PagCLE27.4
PagCLE21.1
PagCLE25.3
PagCLE44.6
PagCLE4.1
PagCLE1
PagCLE3.10
PagCLE3.9
PagCLE6.1
PagCLE14.4.T1
PagCLE4.3
PagCLE27.5
PagCLE20
PagCLE25.9
PagCLE14.1
PagCLE17.3
PagCLE25.5
PagCLE4.4
PagCLE3.4
PagCLE3.8
PagCLE3.5
G01
G02
G03
G04
G05
G06
G08
G09
G10
G11
G12
G13
G14
G15
G16
G17
G19
0 Mb
10 Mb
20 Mb
30 Mb
40 Mb
50 Mb
PagCLE2
PagCLE21.3
PagCLV3.3
PagCLE14.8
PagCLE41.4
PagCLE44.1
PagCLE40.1
PagCLE41.1
PagCLE44.3
PagCLE41.5
PagCLE14.5
PagCLE3.2
PagCLE6.2
PagCLE6.7
PagCLE6.5
PagCLV3.6
PagCLE17.4
PagCLE25.1
PagCLE27.2
PagCLE13.3
PagCLE10.2
PagCLE45.3
PagCLE17.5
PagCLV3.2
PagCLE45.1
PagCLE10.4
PagCLE27.3
PagCLE25.4
PagCLE44.5
PagCLE14.3
PagCLE13.2
PagCLE41.2
PagCLE4.2
PagCLE27.6
PagCLE25.8
PagCLE14.2
PagCLE17.2
PagCLE25.6
PagCLE3.3
PagCLE3.7
PagCLE3.6

Supplementary Figure S2. A, B and C represent the chromosomal location of *CLE* genes on the chromosomes of *P. trichocarpa*, *P. tomentosa*, and *P. alba* × *P. glandulosa*, respectively. Above each colorful bar are the names of chromosomes. The black line indicates the location of the corresponding gene. The vertical scale on the left indicates the size of the chromosome, and the scale represents the Mega Bas

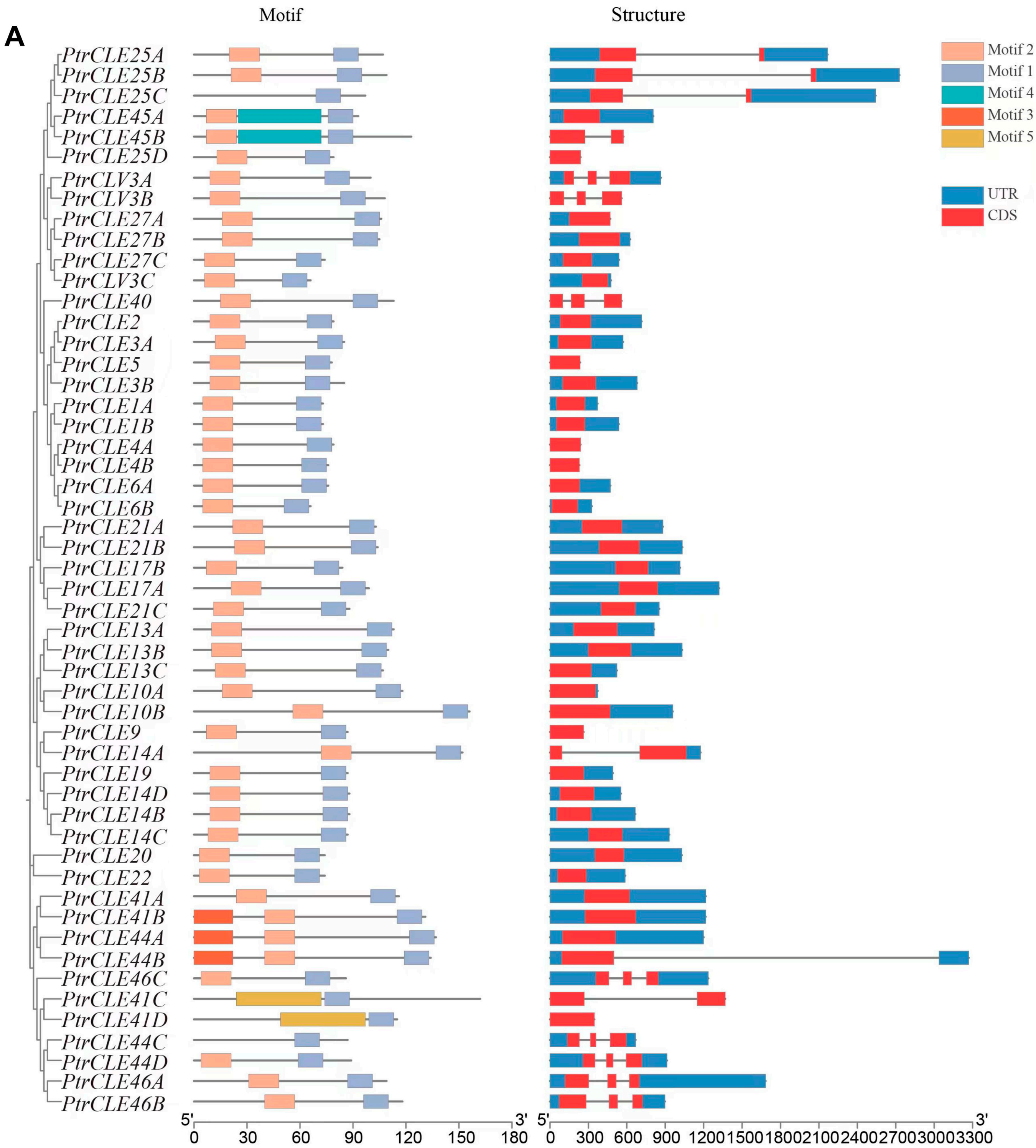
A
Motif
Structure
PtrCLE25A
PtrCLE25B
PtrCLE25C
PtrCLE45A
PtrCLE45B
PtrCLE25D
PtrCLV3A
PtrCLV3B
PtrCLE27A
PtrCLE27B
PtrCLE27C
PtrCLV3C
PtrCLE40
PtrCLE2
PtrCLE3A
PtrCLE5
PtrCLE3B
PtrCLE1A
PtrCLE1B
PtrCLE4A
PtrCLE4B
PtrCLE6A
PtrCLE6B
PtrCLE21A
PtrCLE21B
PtrCLE17B
PtrCLE17A
PtrCLE21C
PtrCLE13A
PtrCLE13B
PtrCLE13C
PtrCLE10A
PtrCLE10B
PtrCLE9
PtrCLE14A
PtrCLE19
PtrCLE14D
PtrCLE14B
PtrCLE14C
PtrCLE20
PtrCLE22
PtrCLE41A
PtrCLE41B
PtrCLE44A
PtrCLE44B
PtrCLE46C
PtrCLE41C
PtrCLE41D
PtrCLE44C
PtrCLE44D
PtrCLE46A
PtrCLE46B
5' 3'
0 30 60 90 120 150 180
5' 3'
0 300 600 900 1200 1500 1800 2100 2400 2700 3000 3300
Motif 2
Motif 1
Motif 4
Motif 3
Motif 5
UTR
CDS

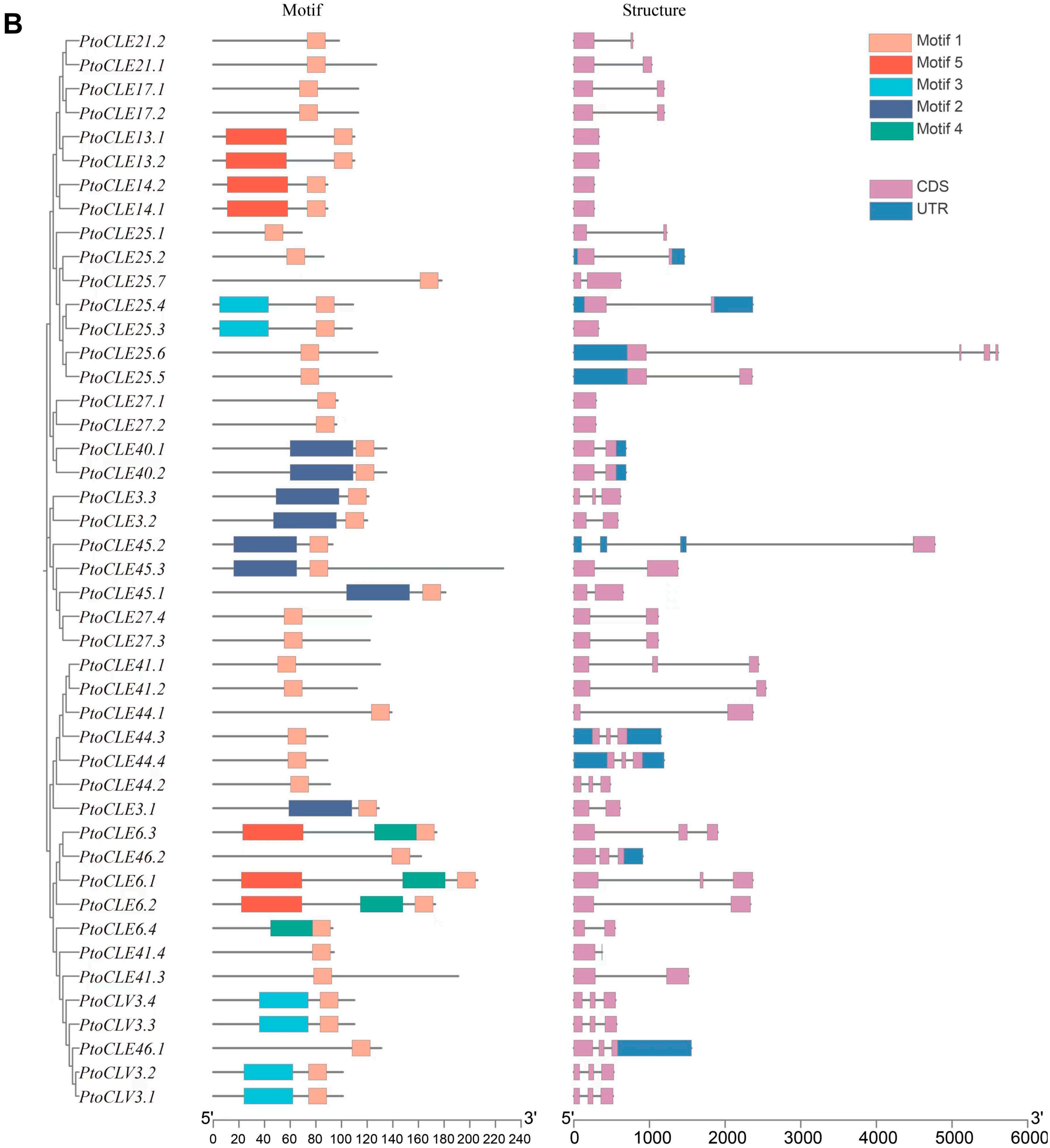
B
Motif
Structure
PtoCLE21.2
PtoCLE21.1
PtoCLE17.1
PtoCLE17.2
PtoCLE13.1
PtoCLE13.2
PtoCLE14.2
PtoCLE14.1
PtoCLE25.1
PtoCLE25.2
PtoCLE25.7
PtoCLE25.4
PtoCLE25.3
PtoCLE25.6
PtoCLE25.5
PtoCLE27.1
PtoCLE27.2
PtoCLE40.1
PtoCLE40.2
PtoCLE3.3
PtoCLE3.2
PtoCLE45.2
PtoCLE45.3
PtoCLE45.1
PtoCLE27.4
PtoCLE27.3
PtoCLE41.1
PtoCLE41.2
PtoCLE44.1
PtoCLE44.3
PtoCLE44.4
PtoCLE44.2
PtoCLE3.1
PtoCLE6.3
PtoCLE46.2
PtoCLE6.1
PtoCLE6.2
PtoCLE6.4
PtoCLE41.4
PtoCLE41.3
PtoCLV3.4
PtoCLV3.3
PtoCLE46.1
PtoCLV3.2
PtoCLV3.1
Motif 1
Motif 5
Motif 3
Motif 2
Motif 4
CDS
UTR
5'
3'
0 20 40 60 80 100 120 140 160 180 200 220 240
5'
3'
0 1000 2000 3000 4000 5000 6000

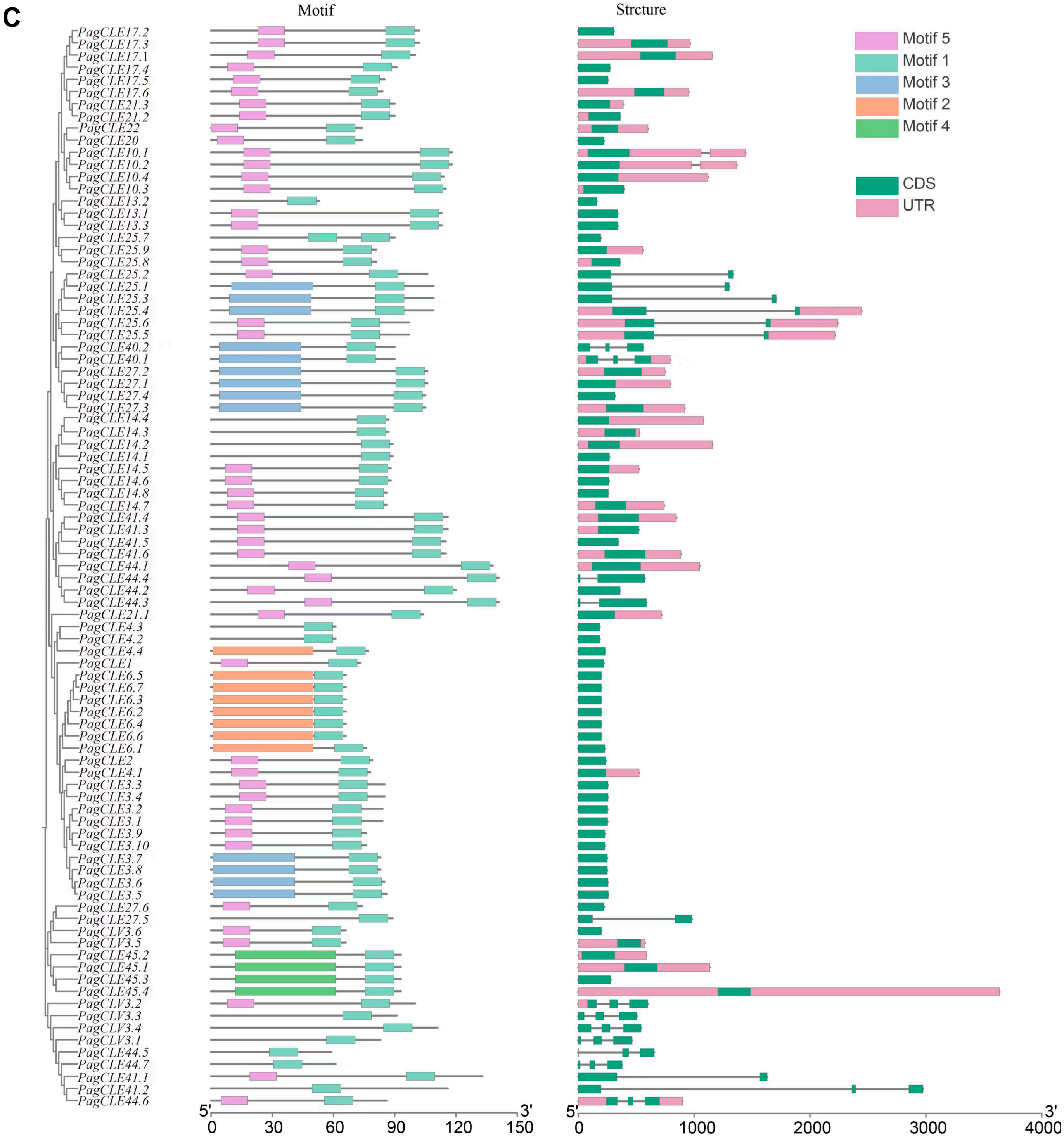

Supplementary Figure S3. A, B and C represent gene structure and conserved motifs of *P. trichocarpa*, *P. tomentosa*, and *P. alba* × *P. glandulosa*, respectively. Five conserved motifs of each poplar were identified. *P. trichocarpa*: motif 1, ASKRLVPSGPBPJHN; motif 2, LLLVLLLJLLLLSSSSTR; motif 3, MDIDPFWITGGWFIITDFNFMAP; motif 4, YGLTSVELILRHDRKAQGTAPQSQRVLKDVDMQGMDTKKSAQANKTFD; motif 5, EEFANRRPDMGNAKTVSKANIIHIPPPSSRRRGRFRAHRSPLPWQEGIF. *P. tomentosa*: motif 1, ASKRKVPSGPBPJHN; motif 2, FLAVVPFSLIFLTSEERILKGGQKAQGTAPNSQRSLKDVDMQKMDTKKSA; motif 3, SGCSTGEKCLYGDAASLVEVKSRKVMVVETGGVRGETTS; motif 4, PLWEARPLDPSAVRRNLIRTIRALGESEAYGDKY; motif 5, AVFITKSLSPLPPSNCRRVRRALEDIEQJLNPEISSTSPLDPRINIAH. *P. alba* × *P. glandulosa*: motif 1, ASKRVVPSGPBPJHN; motif 2, ASLRCYLCVLLIVLSFALYEARPLDPSAVRRNLIRTIRALGESEAVGDQY; motif 3, ASRRAMTSRSLVLVLVVLVLAIFLTSEANIKAGAQALQSEN; motif 4, ICIGFIAVQPDEVYGLTSVELILRHDRKAQGTLPQSQRVLKDVDMQGMDT; motif 5, AVLFLLLJLLVTSL.

I

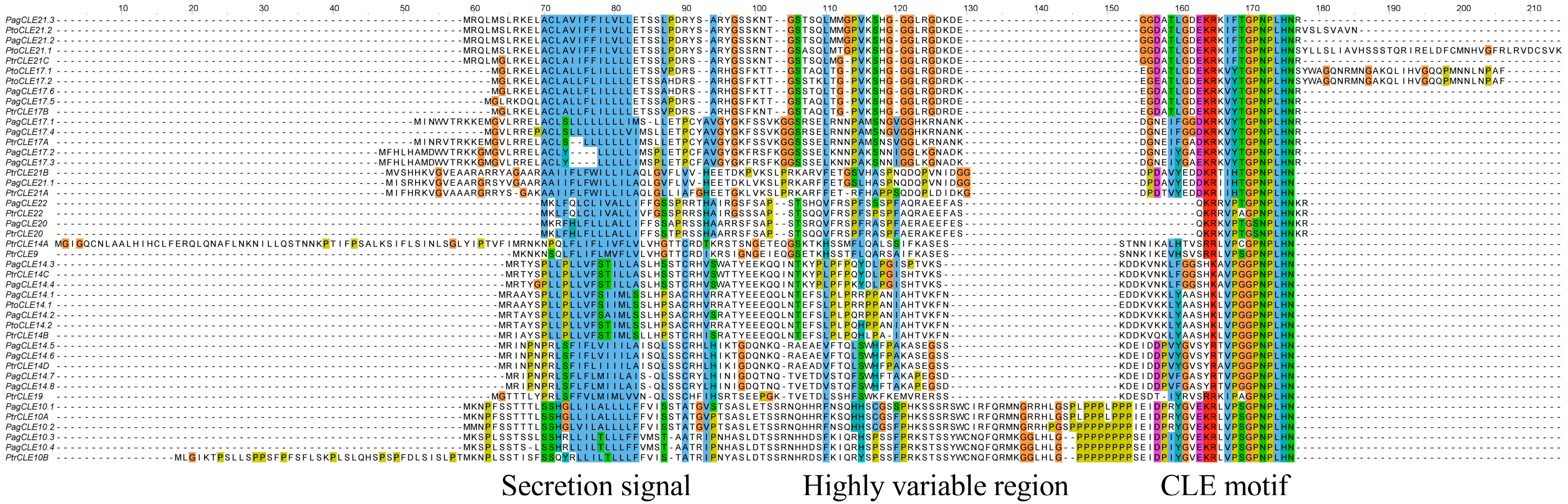
Secretion signal
Highly variable region
CLE motif

II

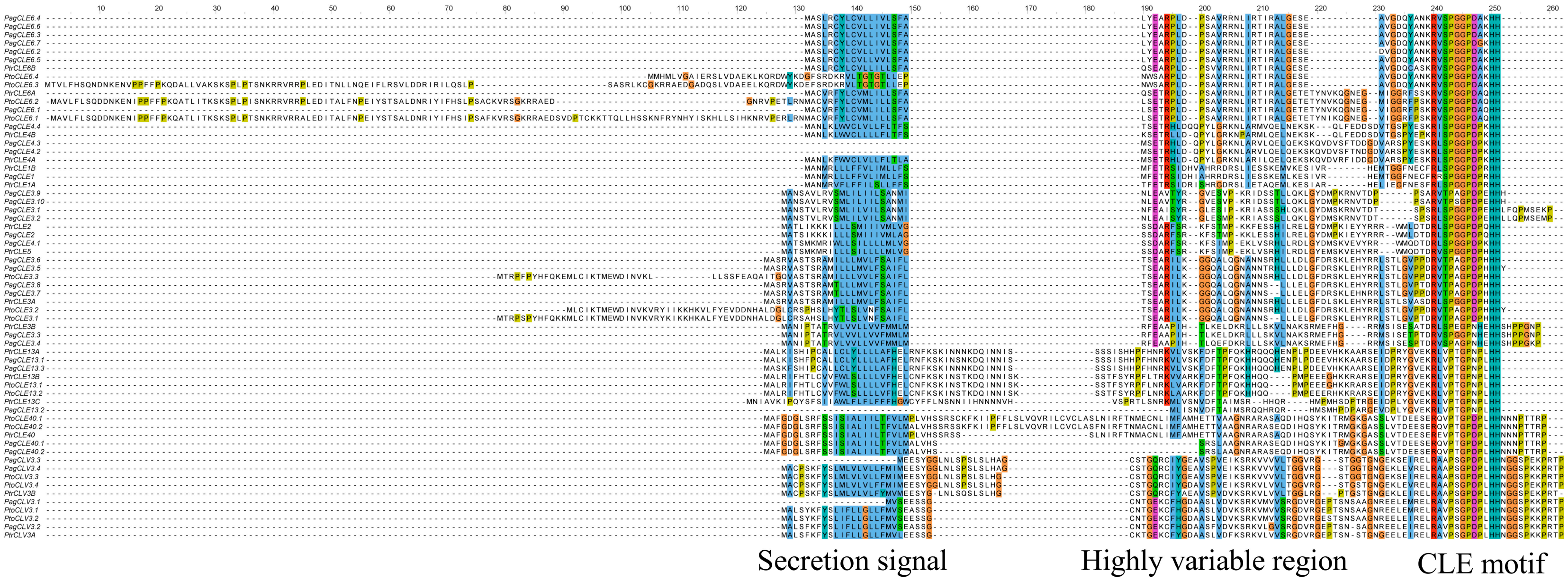
Secretion signal
Highly variable region
CLE motif

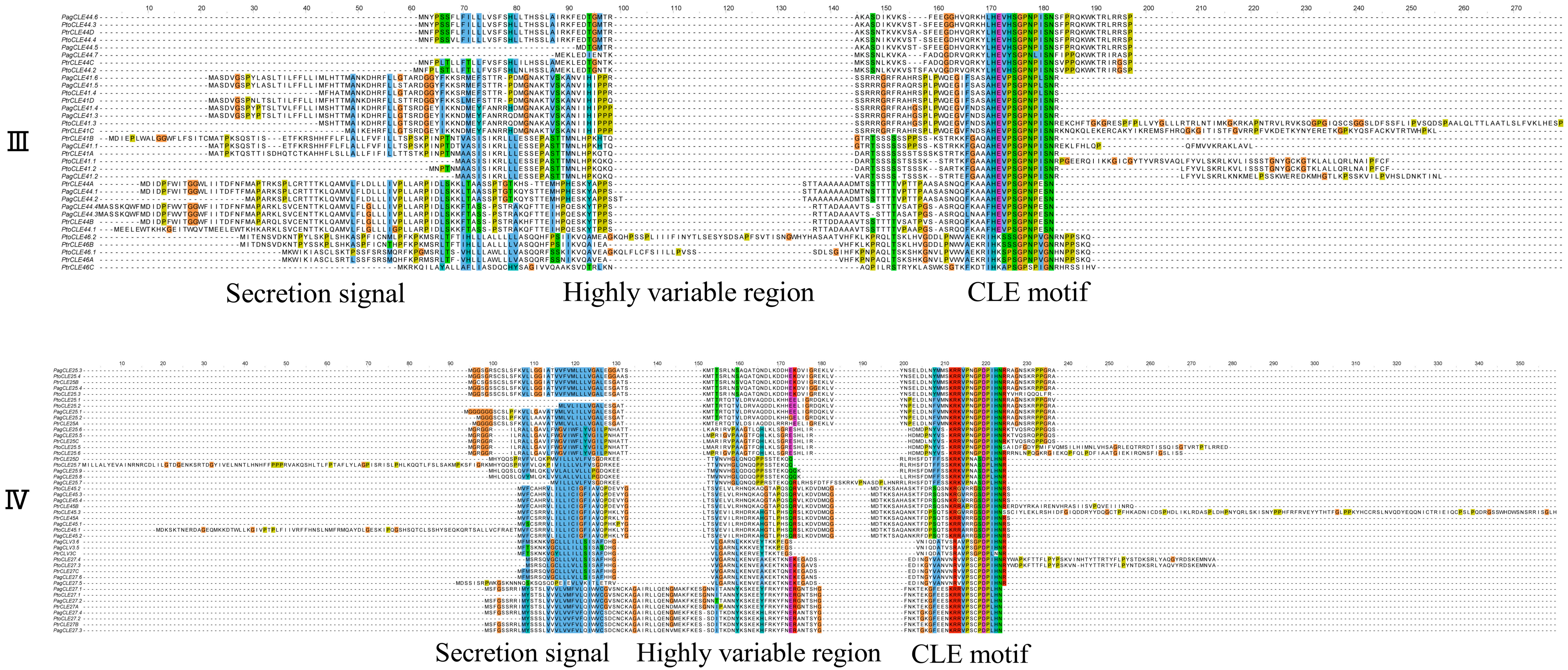

Supplementary Figure S4. Multiple sequence alignment of *P. trichocarpa*, *P. tomentosa* and *P. alba* × *P. glandulosa* CLE pre-propeptides was performed using the ClustalX program. The CLE motif contains 12 conserved cysteines.

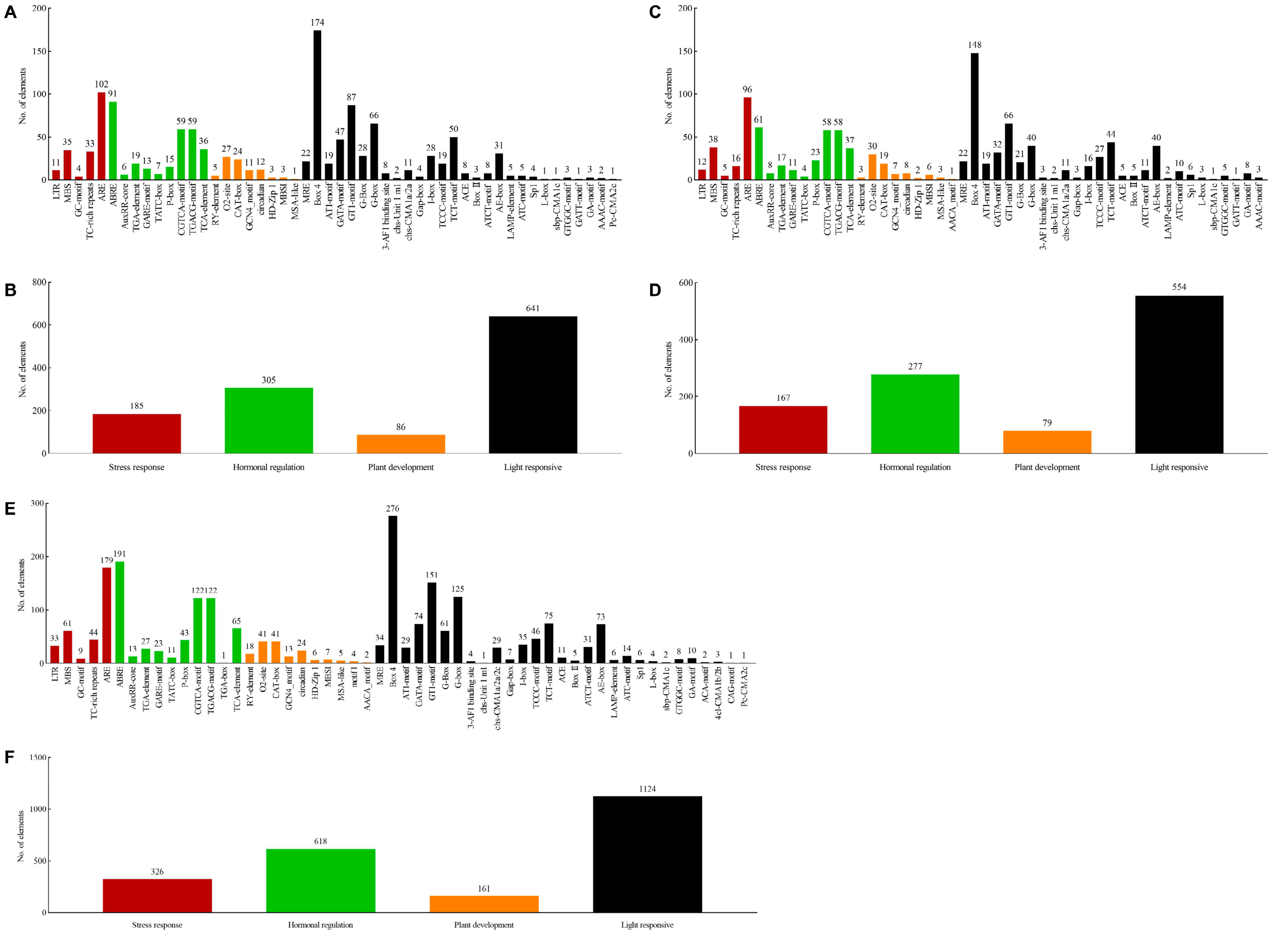

Supplementary Figure S5. The number of each CARE in each poplar species as well as the total amount of each type of CAREs in each poplar species. A and B, *P. trichocarpa*; C and D, *P. tomentosa*; E and F, *P. alba* × *P. glandulosa*.

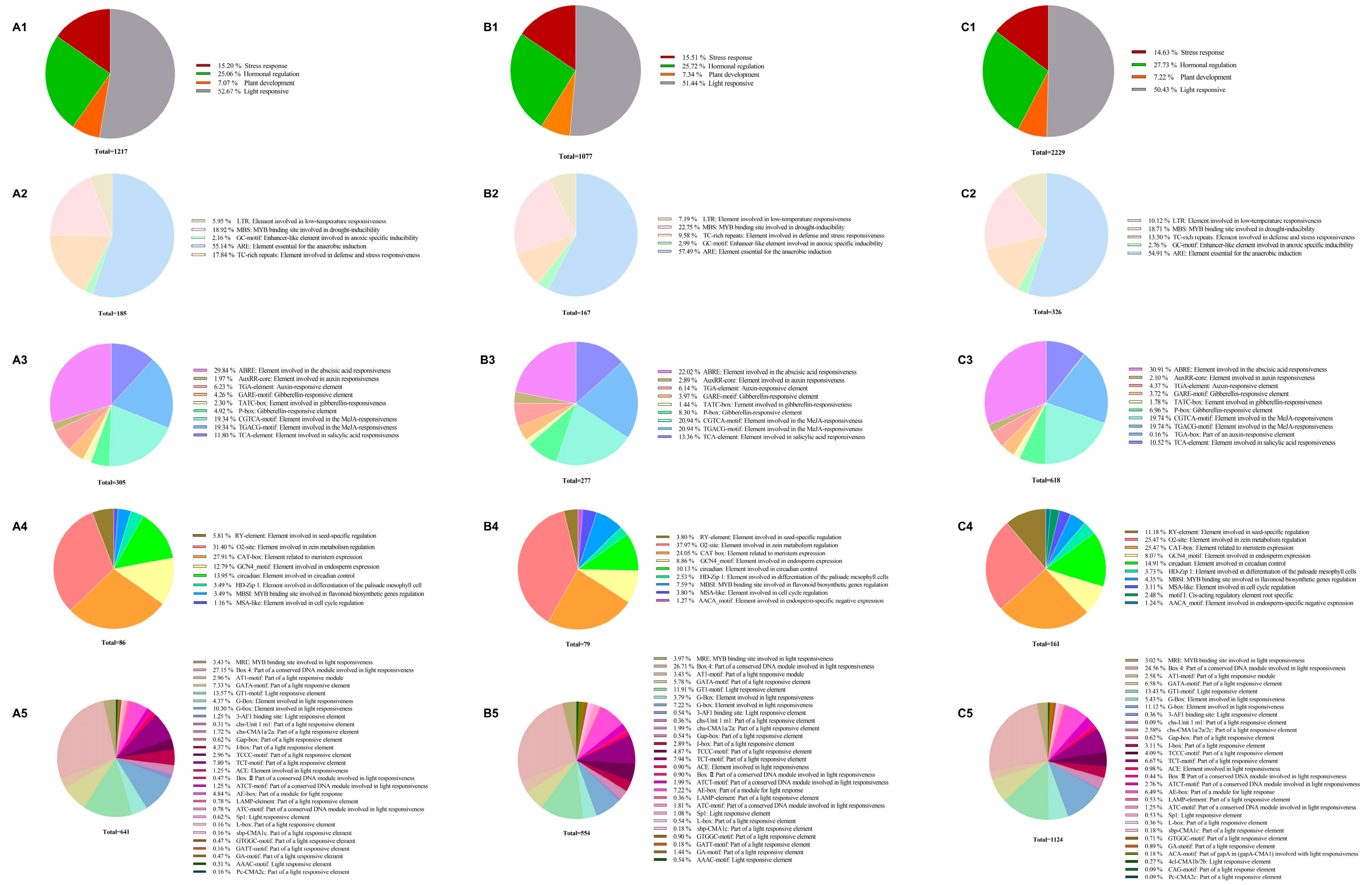

Supplementary Figure S6. The percentage of each type of CAREs in all elements and the percentage of each CARE in each type of elements in the three poplar species. A1-A5, *P. trichocarpa*; B1-B5, *P. tomentosa*; C1-C5, *P. alba* × *P. glandulosa*.

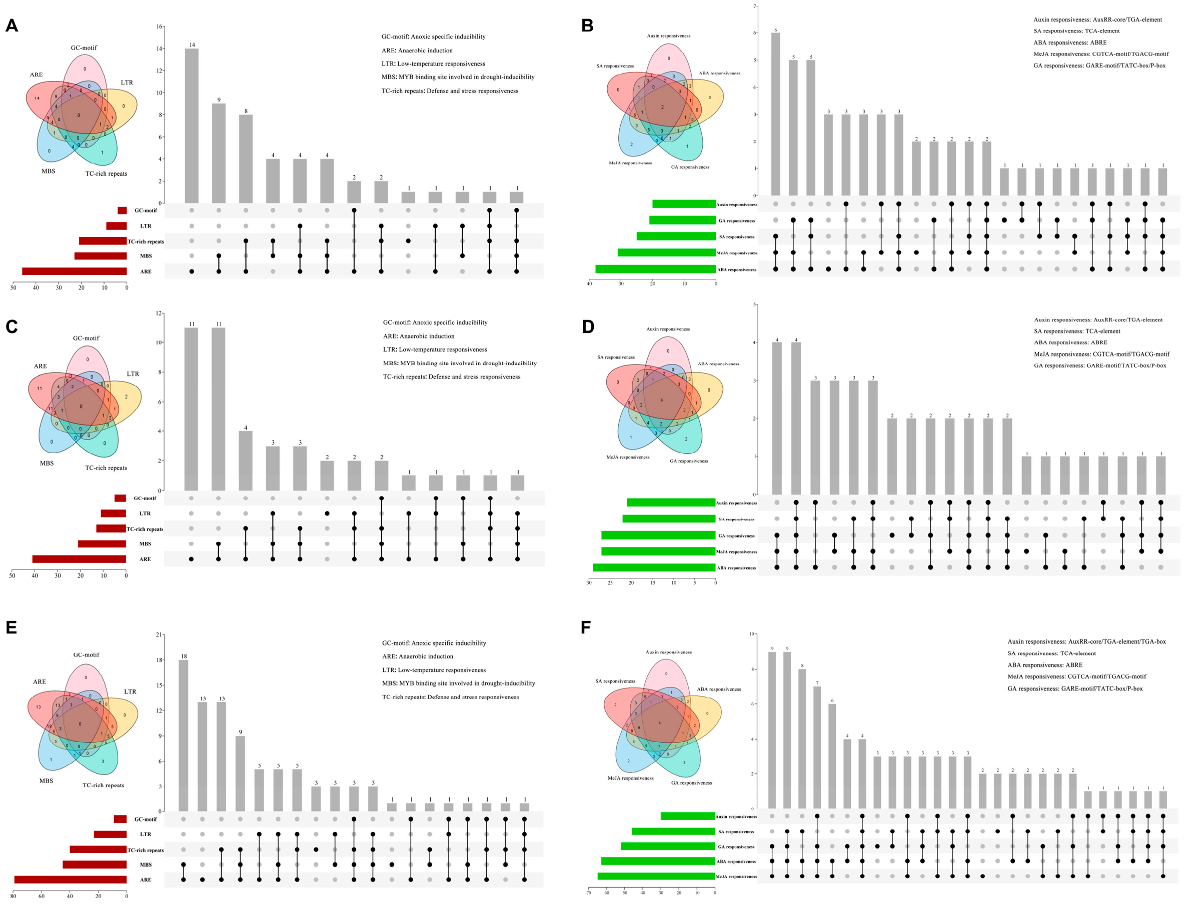

Supplementary Figure S7. Venn diagram and upset diagram illustrating the cis-acting regulatory elements in response to stress and plant hormones at the promoters of CLE genes in P. trichocarpa [A (stress response) and B (hormonal regulation)], P. tomentosa [C (stress response) and D (hormonal regulation)] and P. alba × P. glandulosa [E (stress response) and F (hormonal regulation)]. This figure was generated by the Venn and Upset Plot function of TBtools (https://github.com/CJ-Chen/TBtools).

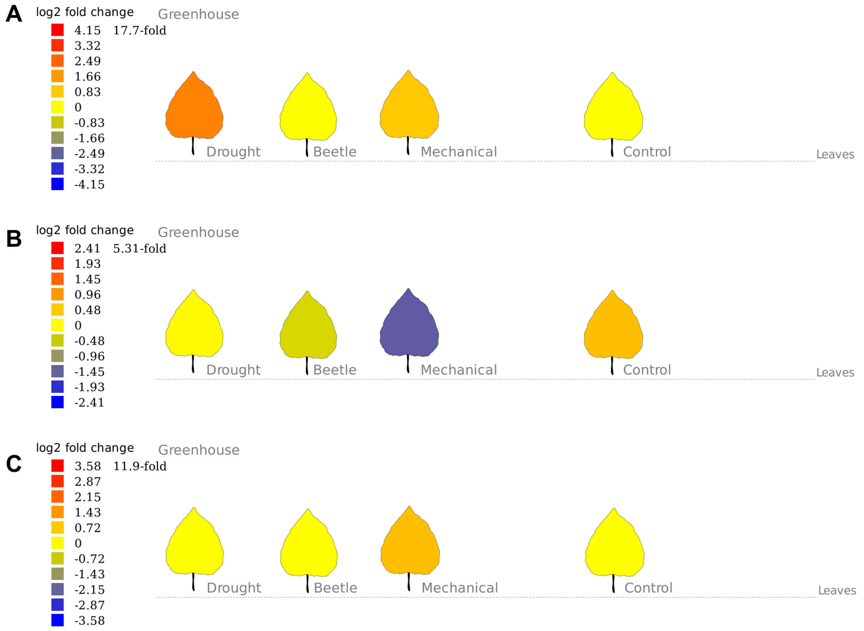

Supplementary Figure S8. The expression patterns of *preCLE13A*, *preCLE13B*, and *preCLE13C* in *P. trichocarpa* leaves under various stress conditions. A, *preCLE13A*. B, *preCLE13B*. C, *preCLE13C*. The data was acquired exclusively from https://plantgenie.org/..

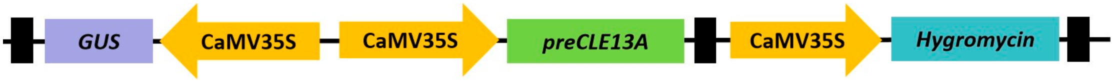

Supplementary Figure S9. Schematic diagram of plant expression vector constructed in this experiment. The small black rectangle represents the NOS terminator.

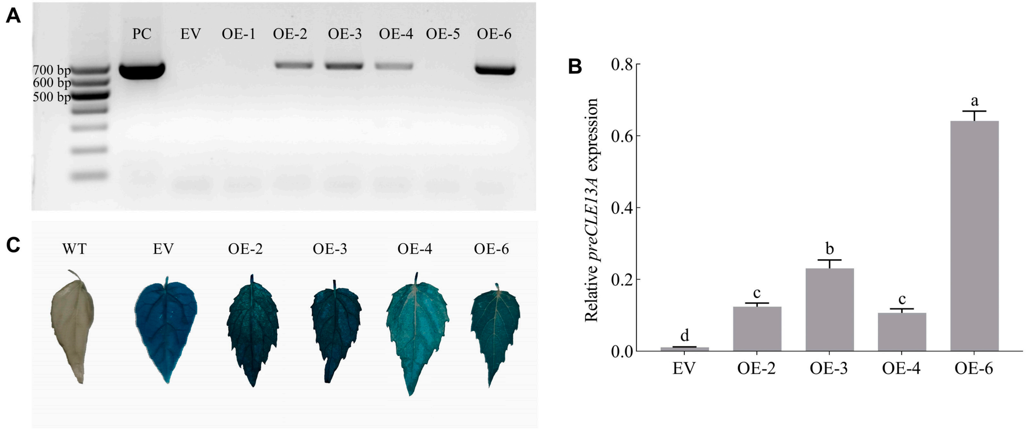

Supplementary Figure S10. Identification of positive *P. tomentosa* lines with overexpression of *preCLE13A* gene. A, The result of polymeric chain reaction to identify whether *preCLE13A* gene is integrated into the genome of *P. tomentosa*. PC, positive control, Here is a plant expression vector containing *preCLE13A* gene. EV, empty vector, Here is the positive *P. tomentosa* plant transformed into empty vector. B, Expression levels of *preCLE13A* in different transgenic lines in (A). C, The GUS reporter gene was successfully expressed in different transgenic lines by staining method. WT, wild type.
